# Lipoxygenase inhibitory synthetic derivatives of methyl gallate regulate gene expressions of COX-2 and cytokines to reduce animal model arthritis

**DOI:** 10.1038/s41598-023-37613-z

**Published:** 2023-06-30

**Authors:** C.S. Sharanya, J. Abhithaj, K.G. Arun, Koti Reddy Eeda, Vignesh Bhat, E.J. Variyar, A. Sabu, M. Haridas

**Affiliations:** 1 Department of Biotechnology and Microbiology and IUCB, Dr Janaki Ammal Campus, Kannur University, Palayad, Thalassery, Kannur, Kerala 670661 India; 2 Transdisciplinary Biology, Rajiv Gandhi Centre for Biotechnology (RGCB), Thiruvananthapuram, Kerala 695014 India; 3 Department of Chemistry, Vignan Foundation for Science Technology and Research, Vignan University (Deemed to be University), Vadlamudi, Guntur, Andhra Pradesh 522 213 India; 4 Department of Chemistry, Mangalore University, Mangalagangothri, Karnataka 574 199 India

**Keywords:** Biochemistry, Biophysics, Biotechnology, Computational biology and bioinformatics, Drug discovery

## Abstract

Mammalian lipoxygenases (LOXs) are involved in the biosynthesis of mediators of anaphylactic reactions and have been implicated in cell maturation, the pathogenesis of bronchial asthma, atherosclerosis, rheumatoid arthritis, cardiovascular diseases, Alzheimer’s disease and osteoporosis. Hence LOX inhibition in chronic conditions can lead to reducing the disease progression, which can be a good target for treating these diseases. The present study deals with designing methyl gallate derivatives and their anti-inflammatory effect by in silico, in vitro and in vivo methods. Designed derivatives were docked against LOX enzyme, and molecular dynamic simulations were carried out. Following the synthesis of derivatives, in vitro LOX inhibition assay, enzyme kinetics and fluorescence quenching studies were performed. One of the derivatives of methyl gallate (MGSD 1) was demonstrated as an anti-inflammatory agent for the treatment of rheumatoid arthritis in the animal model. Amelioration of Freund’s complete adjuvant (FCA)-induced arthritis by methyl gallate and its derivative with a concentration of 10–40 mg.kg^−1^ has been assessed in vivo in a 28-day-long study. *TNF-α* and *COX-2* gene expression were also studied. Methyl gallate synthetic derivatives (MGSDs) inhibited LOX with an IC_50_ of 100 nM, 304 nM, and 226 nM for MGSD 1, MGSD 2, and MGSD 3, respectively. Fluorescence quenching methods also prove their binding characteristics, and 200 ns simulations studies showed that the RMSDs for the entire complex were less than 2.8 Å. The in vivo results showed that methyl gallate was required approximately five times diclofenac for the same level of effect, and the synthesised (MGSD 1) compound required only approximately 1/12 of diclofenac for the same level of effect in in-vivo studies. The preeminent expression of *COX-2* and *TNF-α* genes was significantly decreased after the treatment of the methyl gallate derivative. Hence, the in vivo results showed that the referenced synthetic derivative might have more arthritis-reducing properties than the parent compound methyl gallate and is more potent than the standard drug diclofenac, with no apparent induced toxicity.

## Introduction

Chronic inflammation is the basis of most diseases including tumors, autoimmune disorders, rheumatoid arthritis, cardiovascular diseases, Alzheimer’s disease, and the new pandemic Covid-19. Arthritis is a chronic inflammatory disease of joints that results in synovial hyperplasia with local invasion of bone and cartilage, leading to joint destruction^1,2^. The significant characteristics of arthritis include swelling in joints, joint inflexibility, pannus formation, and synovitis^3^. Millions of people express rheumatoid arthritis (RA), especially in their older age. At the arthritic state, the pro-inflammatory modulators, produced via the stimulation of inflammatory cells in the synovial membrane, are responsible for the augmented bone damage^4^. Furthermore, the migration of leukocytes and other cells in the synovial tissue produces several mediators of inflammation^5,6^. RA pathogenesis is regulated by pro-inflammatory cytokines, interleukin-1 (IL-1) and Tumor necrosis factor-α (TNF-α) and activates a broad array of intracellular signal transduction mechanisms. These pro-inflammatory mediators also increase the expression of cyclooxygenase-2 (COX-2) in synovial tissues by activating transcription factors like nuclear factor- κ B (NF-κB) and c/EBP (CCAAT/enhancer binding proteins). The anti-inflammatory mediators like interleukin-4 (IL-4) and interleukin-10 (IL-10) act antagonistically to combat the inflammatory reactions produced by the pro-inflammatory mediators like TNF-α, interleukin (IL-6), and interleukin-1β (IL-1β)^7,8^.

The development of new therapeutics by exploiting the understanding of the intracellular targets that regulate cytokines is prominent in the current scenario. The transcription factor NF-κB, the major controller of both inflammatory responses and the immune system, is activated in the synovium of patients with RA and regulates genes that contribute to inflammation, including TNF-α, IL-6, interleukin- 8 (IL-8), inducible nitric oxide synthase (iNOS), lipoxygenase-5 (LOX-5) and COX-2. The roles in the pathogenesis of the above-mentioned factors are well documented^9–12^. For example, the activity of NF-κb was inhibited by aspirin and sulfasalazine at high doses^13–15^.

The screening of plants for finding structural leads might be the foundation on synthetic modifications to augment the bioavailability and pharmacokinetics, thus significantly enlightening the efficacy of plant components for therapy. Methyl gallate, is a prominent natural phenolic acid product with good LOX inhibitory potential^16^, found in *Bergenia ligulata* (Wall.) and in many other plants and also shows a heap of natural activities such as anti-inflammatory, antiviral, antimicrobial, and anticancer activities^11,17,18^. Methyl gallate inhibited LOX competitively. However, methyl gallate may interfere with the proper function of the enzyme LOX by competitively binding to its active site and probably to other sites as well. On the contrary, while designing a potent inhibitor should be very specific to the active site. Therefore, this study aims to characterize the efficacy and mechanism of the synthetic derivative of methyl gallate, which is hypothesized to be a potent inhibitor and binds specifically to the active site, in alleviating the disease progression of a chronic inflammatory disease, RA, in terms of LOX, COX-2 and cytokines.

## Results and discussion

This experiment is the continuation of our reported previous study^16^. Methyl gallate, a phenolic compound isolated from *B. ligulata* (Wall.), inhibited LOX enzyme with an IC_50_ of 30.69 µM. The inhibitory properties were analyzed by enzyme assay, kinetics and the binding affinity studies by fluorescence quenching and molecular docking studies^16^. Linoleic and arachidonic acids are the substrates of the enzyme LOX, and their carboxylic acid residues have shown strong interactions with the active site of LOX (Supplementary Fig. 1). Based on the results, we designed new derivatives of methyl gallate (with the incorporation of carboxylic acid groups in ortho, meta, and para positions) and tested them against LOX (Supplementary Fig. 2). Along with this, some other derivatives of methyl gallate were also designed. In the virtual screening studies, high and better glide scores (− 9 kcal/mol to − 11 kcal/mol) were obtained for carboxylic acid-containing (MGSD 1–3) compared to other derivatives, which were then selected for further studies.

### E-pharmacophore based screening and designing of MGSDs

E-pharmacophore based studies are new to the drug discovery process, where we can screen large numbers of designed libraries in a brief period with minimum computational facilities. It is a collaboration of both electronic and steric features necessary for the ideal supramolecular interactions with a specific biological target and to trigger (or block) its biological response.

In our study, an e-pharmacophore hypothesis was generated to screen the potential LOX inhibitor using its crystal structure complex with nordihydroguaiaretic acid (NDGA, PDB ID: 6N2W). The bound inhibitor, nordihydroguaiaretic acid (NDGA) interacts strongly with the active site amino acid residues (Supplementary Fig. 3). Energy optimized, seven-featured pharmacophore hypothesis, AARRRDN was obtained based on the ligand–protein complex. The generated e-pharmacophore model contained two hydrogen bond acceptors (A), three aromatic rings (R), hydrogen-bond donor (D) and negatively ionizable (N) group. The distance between each pharmacophoric feature and their planar representation is shown in the figure (Supplementary Fig. 4). The hypothesis, AARRRDN was used for screening potential molecules in the designed compounds with comparable pharmacophore features (Supplementary Fig. 5). During the screening, the phase module analyses the fitness of compounds with the query hypothesis and ranks the compounds based on the fitness score (Table 1).

**Table 1:** Interaction details of methyl gallate and MGSDs.

Inhibitors	Structure	Fitness score	Glide score in kcal/mol	No. of H bonds	Interacting residues
Methyl gallate		1.311	− 6.75	1	Tyr558
MGSD 1 [(2,2′,2″-((5-carboxybenzene-1,2,3-triyl)tris(oxy)) tri acetic acid]		1.461	− 11.61	5	Phe177
					Leu179
					Asn180
					Gln413
					Gln557
MGSD 2 [2,2′-((5-carboxy-3-hydroxy-1,2-phenylene)bis (oxy))diacetic acid]		1.421	− 10.32	2	Phe177
					Lys409
MGSD 3 [(3-(carboxymethoxy)-4,5-dihydroxybenzoic acid]		1.372	− 9.02	4	Ile406
					Phe177
					Leu179
					Asn180
NDGA		0.977	− 7.69	3	Ile406
					Asn554
					Ala672

Later, molecular docking was carried out with these ranked molecules. Extra precision (XP) docking method was applied for the docking studies and inferred that the methyl gallate derivatives had a glide score which was higher than that of parent molecule methyl gallate (Table 1). The carboxylic derivatives 2,2′,2″-((5-carboxybenzene-1,2,3-triyl) tris (oxy)) tri acetic acid (MGSD 1), 2,2′-((5-carboxy-3-hydroxy-1,2-phenylene) bis (oxy)) diacetic acid (MGSD 2), (3-(carboxymethoxy)-4,5-dihydroxybenzoic acid (MGSD 3) showed a glide score of − 11.61, − 10.32, and − 9.02 kcal/mol respectively. The docking procedure, validated by redocking the crystallographic ligand with LOX and the superimposed image of both docked and crystal structure of NDGA is shown in the figure (Supplementary Fig. 6). During the redocking analysis, RMSD value was found to be 0.36 Å and the generated poses correctly positioned the ligand at the active site. The G score obtained was compared with screened ligands. NDGA got a score of − 7.69 kcal/mol and the screened derivatives had scores greater than that of NDGA (Table 1).

In the LOX structure, the non-heme iron in the catalytic site is encircled by conserved histidine residues (His367, His372 & His550). Furthermore, a conserved helix, which armours the catalytic iron in sLOX-1^19^ is present in the human LOX also. In this bowed helix, there is a Leu414 which is at the apex of this helix anticipated to regulate the access of oxygen to the substrate or to place the substrate for an attack. In addition to this, the other amino acids which are essential for the catalytic site are Leu420 and Phe421. Both the α-helix and α2- helix coordinate the active site, whereas the α2-helix present at the one edge of the active site, is a three-turn helix bordered by stretched loops. α2-helix contains specific amino acids Phe177 and Tyr181 which make the catalytic iron inaccessible and yields its distinctive active site cavity. Ala603, Ala606, His600, Thr364 is required for the specific activity of Phe177 and Tyr181 which make a "corks" like structure (FY cork) at one end of the cavity^20,21^.

The catalytic site is elongated and does not have a clear access to bulk solvents, lined with invariant amino acids. The major amino acids which are conserved in all AA (arachidonic acid)-metabolizing LOXs are Leu368, Leu373, Leu414 and Leu607 and Ile406. These amino acids form a hydrophobic envelop where the pentadiene must be placed for catalysis. The amino acid residue Trp599 appears to support the FY cork from one side^21^. Asn407 and His432 also help to define the active site. The mechanism of substrate entry and action is similar to that of plant LOX and the only difference is in the positions of amino acids in certain places.

**Figure 1:**
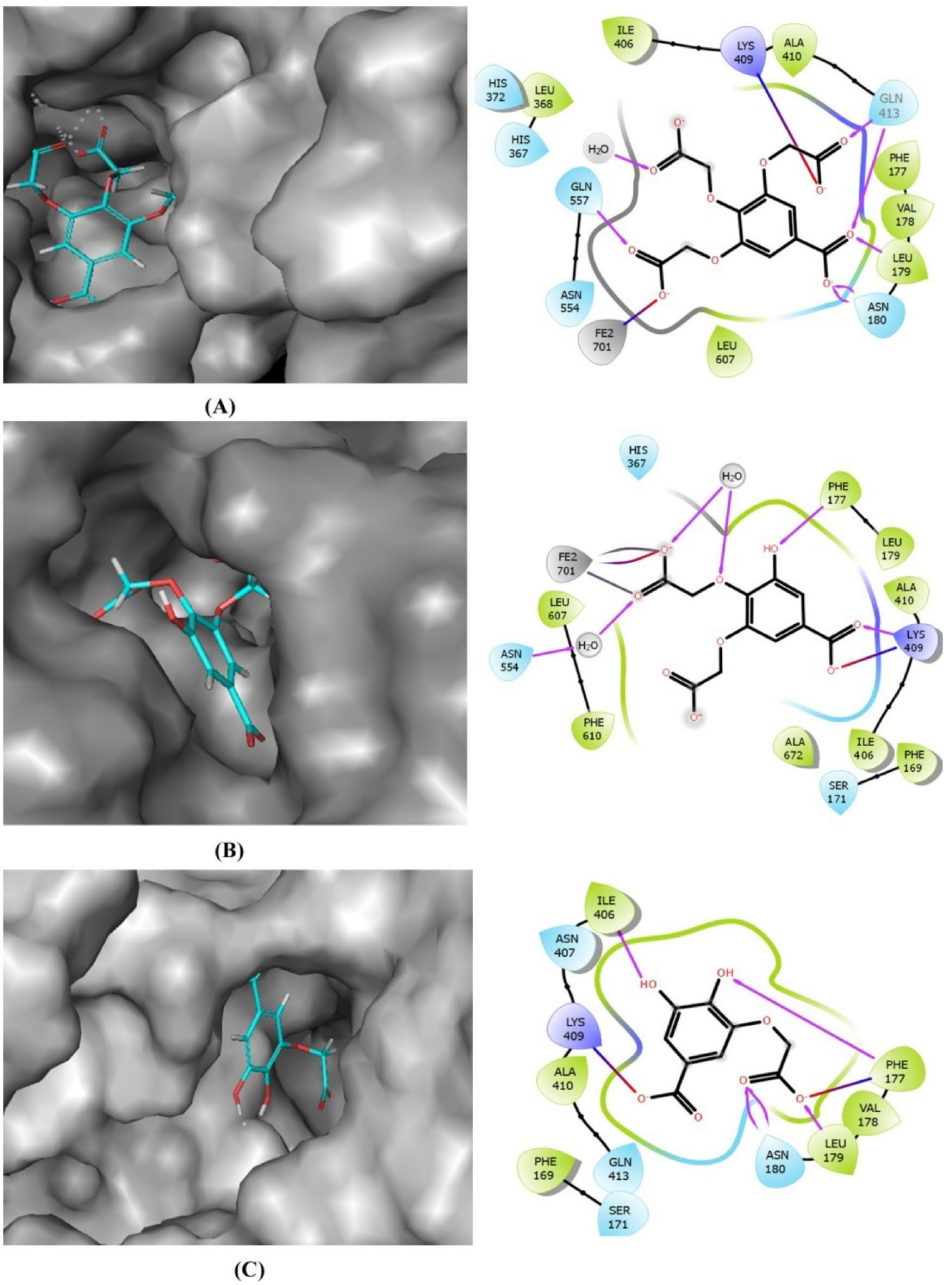
Interaction of MGSDs [MGSD 1 (**A**), MGSD 2 (**B**), MGSD 3 (**C**)] at the active site of LOX. The hydrogen bond interactions were shown by purple color bonds, and the red-blue combination indicated the salt bridge formations.

The compounds got perfectly docked at the active site of LOX and made hydrogen bond interactions with the residues Phe177 and Gln557. All the hydrogen bonds and van der Waals interactions stabilized the protein–ligand interactions. MGSD 1 formed six hydrogen bonds with active site residues Leu179, Asn180, Gln413 and Gln557, also formed salt bridge with catalytic iron and Lys409. Likewise, MGSD 2 also formed hydrogen bond interactions with catalytic residues Phe177 and Lys409 and salt bridge with catalytic iron and Lys409. In the case of MGSD 3, hydrogen bonding was found between Phe177, Leu179, Asn180 and Ile406. Salt bridge was formed between Lys409 and Phe177 (Fig. 1).

**Figure 2:**
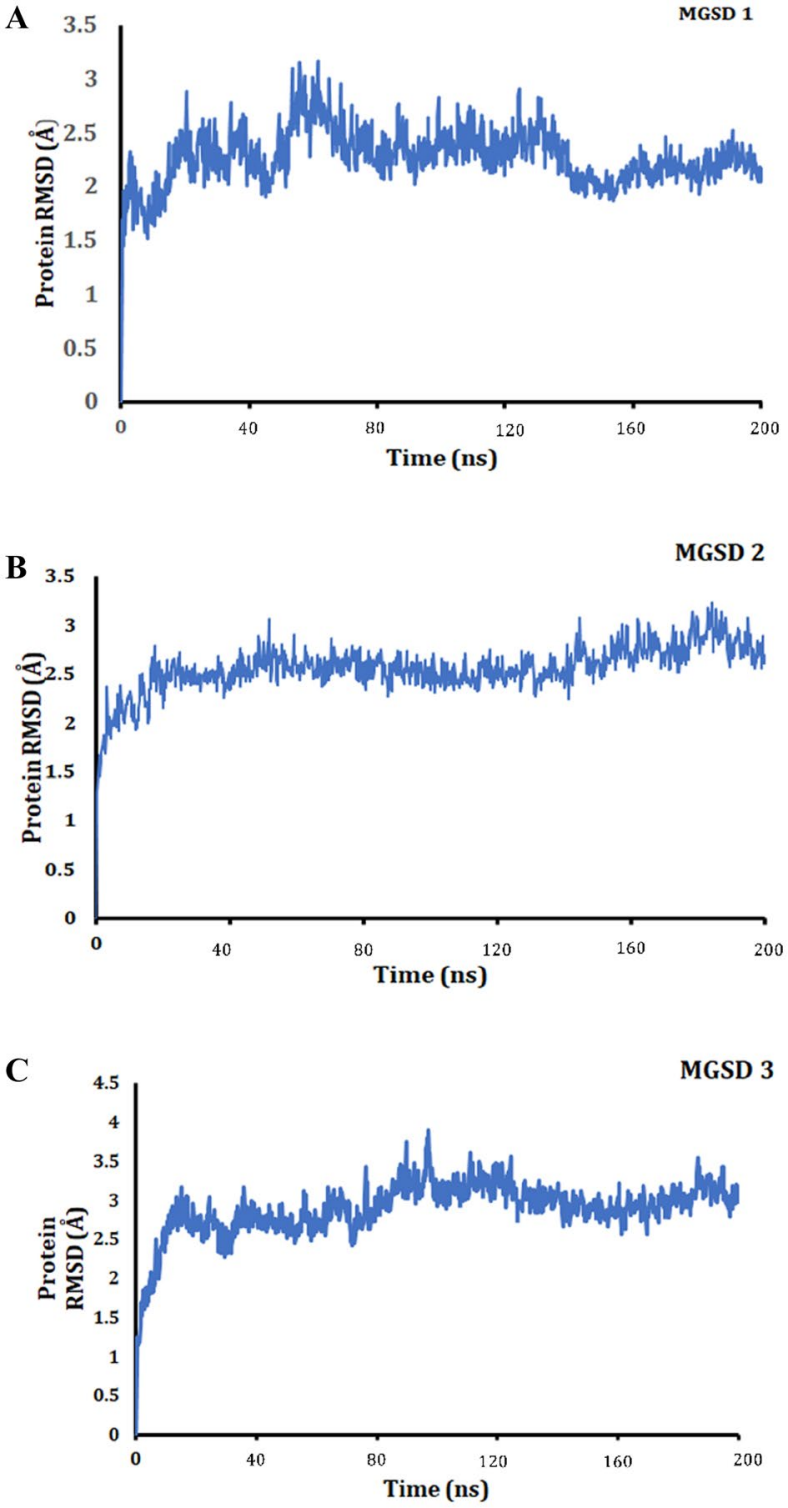
A plot of RMSD versus time during 200 ns MD simulation of the LOX with MGSDs [MGSD 1(**A**), MGSD 2 (**B**), and MGSD 3 (**C**)].

**Figure 3:**
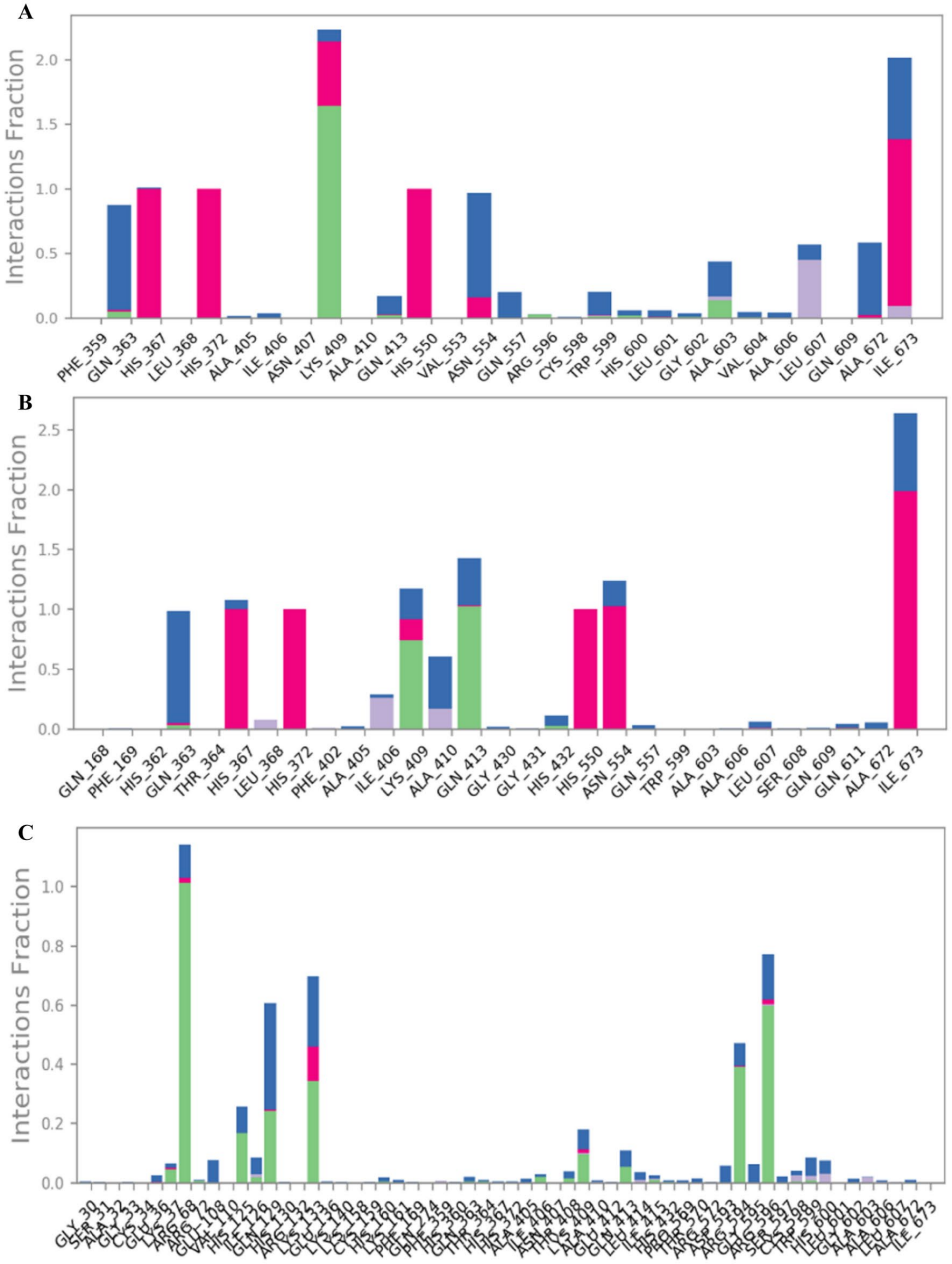
Interaction maintained throughout the 200 ns simulation [MGSD 1(**A**), MGSD 2 (**B**) and MGSD 3 (**C**)]. The different color in the bar diagram indicates the different type of interaction. (Green color indicates the hydrogen bond interaction, purple color indicates the hydrophobic interaction, red color indicates the ionic interaction, and the blue color indicates the water bridges).

The stability of protein–ligand interaction was further studied by molecular simulation. It was seen that the complex structures were balanced out within around the 200 ns simulation. The LOX- MGSD 1 complex had an RMSD below 2.5 Å and MGSD 2 and MGSD 3 showed an RMSD below 3.5 Å. No extreme structural abnormalities were observed with derivatives in their binding positions even after 200 ns MD simulation in the existence of explicit water molecules. It was seen that the coordinate and hydrogen bonds were conserved throughout the simulation (Figs. 2 and 3). The outcomes recommended that the binding of this compound was firm at their binding position.

The Qikprop module analyzes the pharmacokinetic property of the designed molecules. The values of the synthetic compounds came under the recommended range of the descriptor (Table 2).

**Table 2:** Pharmacokinetic properties predictions of MGSDs predicted using the QikProp module of Schrodinger suite.

Compound ID	#stars^a^	#rotor^b^	SASA (Å)^c^	Donor HB^d^	Accept HB^e^	QPlogPo/w^f^	QPlogKp^g^	Rule of Five^h^	Rule of Three^i^
MGSD 1	4	10	572.735	4	10.25	0.268	– 8	1	1
MGSD 2	2	8	496.541	4	8.25	0.003	− 7.25	0	1
MGSD 3	0	6	426.442	4	6.25	− 0.31	− 6.56	0	1

^a^Number of descriptor values fall outside values of 95% known drugs (recommended range: 0 to 5).

^b^Number of rotatable bonds (recommended range: 0–15).

^c^Total solvent accessible surface area (SASA) in square angstroms (recommended range: 300 to 1000).

^d^Number of hydrogen-bond donors (recommended range: 0 to 6).

^e^Number of hydrogen-bond acceptors (recommended range: 2 to 20).

^f^Predicted octanol/water partition coefficient (recommended range: – 2 to 6.5).

^g^Predicted skin permeability (recommended range: – 8.0 to – 1.0).

^h^Rule of five: Lipinski’s rule of five (Maximum is 4).

^i^Rule of three: Jorgensen’s rule of three (Maximum is 3).

### Synthesis

**MGSD 1:** ^**1**^**H NMR** (400 MHz, DMSO-*d6*): δ 12.93 (bs, 3H), 7.14 (s, 2H), 4.78 (s, 4H), 4.66 (s, 2H); **LCMS:** *m/z* calculated for C_13_H_12_O_11_: 344.04; Observed mass: 344.90 (M + 1); CHN analysis (C, 47.46; H, 3.76) (Supplementary Figs. 7 and 11; Appendix I).

**MGSD 2:** ^**1**^**H NMR** (400 MHz, DMSO-*d6*): δ 7.13 (s, 1H), 6.99 (s, 1H), 4.87 (s, 2H), 4.67 (s, 2H); **LCMS:** *m/z* calculated for C_13_H_12_O_11_: 286.03; Observed mass: 287.22 (M + 1); CHN analysis (C, 46.28; H, 3.63) (Supplementary Figs. 8, 9 and12; Appendix I).

**MGSD 3:** ^**1**^**H NMR** (400 MHz, DMSO-*d6*): δ 7.11 (s, 1H), 6.93 (s, 1H), 5.82 (bs, 1H), 5.59 (s, 1H), 4.73 (s, 2H); **LCMS:** *m/z* calculated for C_9_H_8_O_7_: 228.03; Observed mass: 227.26 (M-1); CHN analysis (C, 45.39; H, 3.45) (Supplementary Figs. 10 and13; Appendix I).

### In vitro LOX assay and Michaelis–Menten enzyme kinetics

In vitro LOX inhibitory effects of MGSDs are depicted in the Supplementary Fig. 14. The three derivatives of methyl gallate with 20 μM concentration showed higher inhibition than the parent compound methyl gallate at 0.2 mM. Methyl gallate showed an inhibition rate of 61 ± 1.35% whereas MGSD 1, MGSD 2 and MGSD 3 had inhibitory profiles of 70 ± 3.45%, 68 ± 4.32% and 65 ± 2.65% of inhibition respectively.

**Figure 4:**
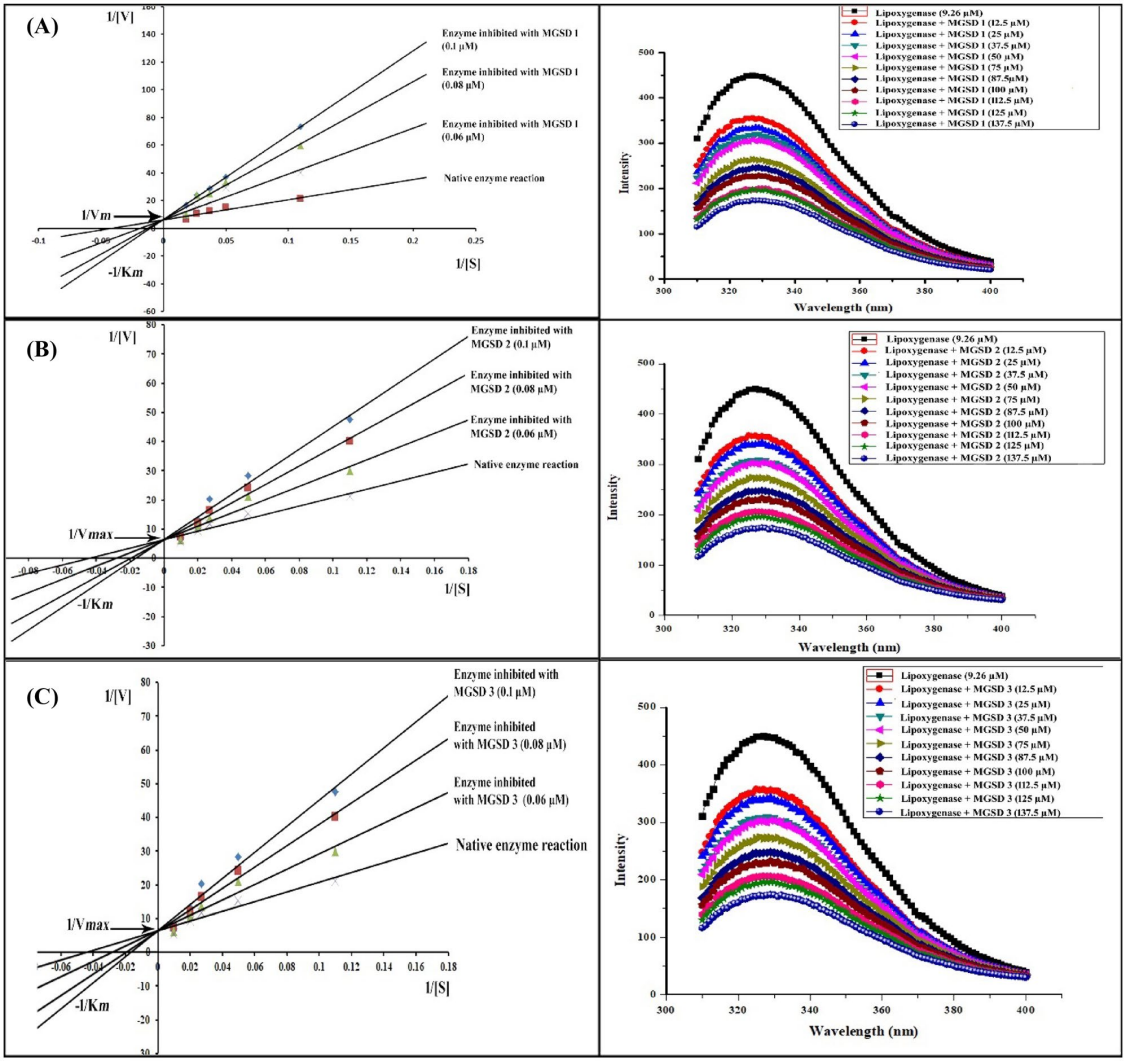
The Line weaver Burk plot and steady-state fluorescence spectra of LOX—MGSD 1 (**A**), MGSD 2 (**B**), and MGSD 3 (**C**) complexes.

Since the compounds of study were confirmed to be LOX inhibitors, enzyme kinetics studies were carried out to establish the mode of inhibition. From the Line-weaver-Burk plot (Fig. 4) the mode of inhibition of MGSDs was identified as competitive. The plots of 1/[V] versus 1/[S] intersected on Y-axis at V_max_ of 0.2 µM/mg/min. Since the nature of inhibition was found to be competitive, V_max_ remained unchanged and K_*m*_ found to be altered. The K_*i*_ of each compound was found to be 0.03 µM, 0.1 µM, and 0.06 µM. Regarding K_*i*_*,* the IC_50_ values also were found to be 0.102, 0.34 and 0.226 µM for MGSD 1, MGSD 2 and MGSD 3respectively.

### Fluorescence quenching studies

Competitive inhibition relies on the referenced molecule displacing linoleic acid from the active site of the enzyme and as a consequence, the site of interaction of derivatives at LOX should be located near the catalytic center. To verify this point and to identify the binding sites and binding affinity of MGSDs with LOX, the quenching studies of intrinsic fluorescence of Tyr and Trp residues of LOX by MGSDs were performed. Fluorescence emission spectra of LOX in the presence of MGSDs at various concentrations were shown to be low (Fig. 4). When LOX was excited at 280 nm broadband emission with a maximum at 333 nm corresponding to Tyr and Trp were detected. By adding MGSDs with increasing concentrations into LOX, the fluorescence intensity of LOX decreased progressively indicating their complex formation with lipoxygenase and led to quenching of intrinsic fluorescence of MGSDs.

To analyze the type of mechanism, the data of fluorescence of LOX was plotted, as relative fluorescence intensity (F_0_/F), against MGSDs. Here F and F_0_ are the fluorescence intensity in the presence and absence of MGSDs respectively. The Stern–Volmer constants were obtained from the plot by linear regression using the expression:

$${\text{F}}_{0} /{\text{F }} = { 1 } + {\text{ Ksv}}\left[ {\text{Q}} \right].$$

Ksv is the Stern–Volmer constant and [Q] is the respective concentration of derivatives. Ksv which depicts the binding affinity between the fluorophore (protein) and quencher (ligand), were obtained from the slopes of the plot (Supplementary Fig. 15). The binding affinity of the compounds were shown in the table (Table 3) and the increasing affinity in the order MGSD 1 < MGSD 2 < MGSD 3. The Fluorescence quenching data provided information about the binding constant (K), the number of binding sites (n), and the values could be arrived by applying the equation.

$${\text{Log F}}_{0} - {\text{F}}/{\text{F}} = {\text{ log K }} + n\,\,{\text{log}}\,\left[ {\text{Q}} \right].$$

**Table 3:** The binding constants and the number of binding sites in LOX—MGSDs.

Details of the compounds	Quenching constant (Ksv)	Number of the binding site (n)	Binding constant (K)
MGSD 1	6.2 × 10^3^ M^−1^	1.05	1.33 × 10^3^ M^−1^
MGSD 2	6.3 × 10^3^ M^−1^	1.4	2.28 × 10^3^ M^−1^
MGSD 3	7.8 × 10^3^ M^−1^	1.4	2.06 × 10^3^ M^−1^

Supplementary Fig. 16 shows the plot of log F_0_–F/F versus log [MGSDs]. The number of binding site/s (n) and binding constant (K) were obtained from the slope and Y-intercept respectively and are shown in the Table 3. The number of the binding site obtained was in between 1 and 1.4, which means the presence of a single binding site in LOX for the drugs. The correlation coefficient values obtained were indicative of the drug interaction with LOX enzyme.

### In vivo anti-inflammatory effects of methyl gallate and its derivative MGSD 1

The in silico (glide score and molecular dynamics simulation) and in vitro (enzymatic and kinetic) results suggest that MGSD 1 has greater potential than methyl gallate and other MGSDs. To further confirm the efficacy of MGSD 1 in the in vivo model, it was subsequently tested against FCA-induced arthritis as a model of chronic inflammation.

#### Acute toxicity

**Scheme 1:**
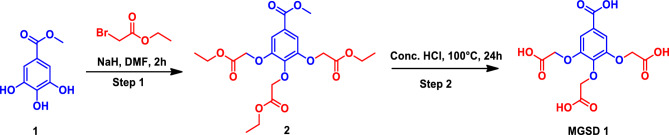
Synthetic scheme of MGSD 1.

According to the OECD guideline 420, the toxicity of the compounds was analysed in female Sprague Dawley rats. The female rats used for the study were nulliparous and non-pregnant. Female rats are used conventionally since they are generally more sensitive than males. At the initial period, they were found to be at an age of 8–12 weeks old. The compounds were dosed at 5, 50, 100, 300 mg.kg^–1^ and found to be stable and didn't cause any toxicity/mortality in the experimental rats. The groups of animals of a single sex (female) are dosed in a stepwise procedure using the fixed doses of 5, 50, 300 and mg.kg^–1^ as per OECD guideline 420. The initial dose level was selected on the basis of a sighting study as the dose expected to produce some signs of toxicity without causing severe toxic effects or mortality. Clinical signs and conditions associated with pain, suffering, and impending death, are described in detail in a separate OECD Guidance Document^22^. Further groups of animals were dosed at higher or lower fixed doses, depending on the presence or absence of signs of toxicity or mortality. This procedure was continued until the dose causing evident toxicity or no more than one death was identified, or when no effects were seen at the highest dose or when deaths occured at the lowest dose. Animals were observed individually after dosing at least once during the first 30 min, periodically during the first 24 h, with special attention given during the first 4 h, and daily thereafter, for 14 days. In the present study, the rats were healthy, and the experiments were continued (Scheme 1).

**Scheme 2:**
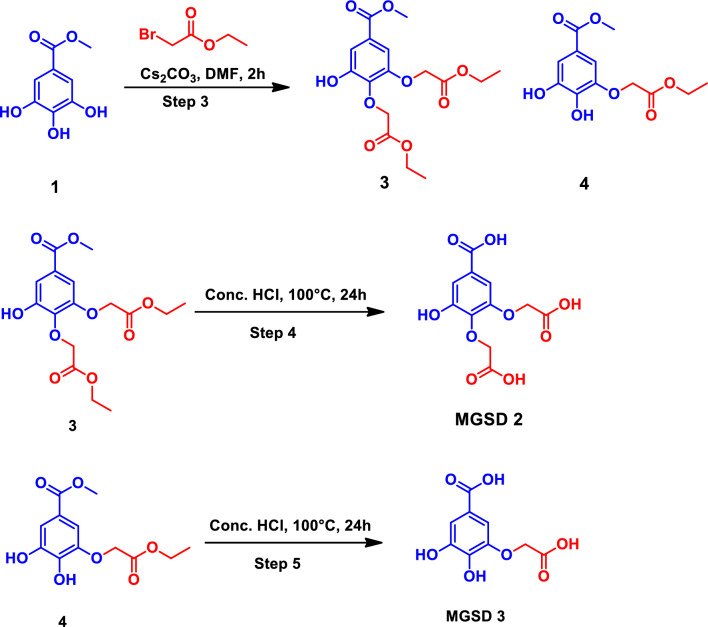
Synthetic scheme of MGSD 2 and MGSD 3.

Based on these observations, two doses, 20 mg.kg^–1^and 40 mg.kg^–1^ for methyl gallate and 1 mg.kg^–1^ and 10 mg.kg^–1^ for MGSD 1 were selected and administered for further experiments. The low and high dose for methyl gallate is 20 and 40 mg.kg^–1^; whereas MGSD 1 low and high dose is 1 and 10 mg.kg^–1^. The standard drug diclofenac is used at a concentration of 10 mg.kg^–1^. So, we used MGSD 1 at this concentration, and methyl gallate, a herbal compound, was used higher than the synthetic compounds. That’s why we have taken different concentrations for methyl gallate and MGSD 1. Diclofenac is a known drug used for chronic inflammation, and the dosage is 10 mg.kg^–1^. So, MGSD 1 was used at this concentration. Since methyl gallate is a herbal compound, it was used at a higher concentration than the synthetic compounds. The authors try to convey that the time taken by the herbal compound was slightly longer than synthetic compounds, hence, the different concentrations for methyl gallate and MGSD 1. The concentrations were determined based on preliminary experiment (Scheme 2)

.

#### Induction of chronic inflammation by FCA

Dysregulated inflammation plays a central role in the case of diverse diseases. Prolonged inflammatory responses, affecting the cells at the site of inflammation are the crucial components of chronic inflammation. During the chronic condition there occur continuous destruction and repair of cells. Chronic inflammation could be induced by Freund's Complete Adjuvant containing a suspension of heat-killed *Mycobacterium tuberculosis* (10 mg/mL; 100 µL) in the vehicle. The major response was the inflammation at the site of injection and there was a migration of leukocytes and they interacted with the antigen. The reactions resulted in granuloma at the site of injection and structural changes in the lymph node^23–26^.

#### Hind paw volume measurement

**Figure 5:**
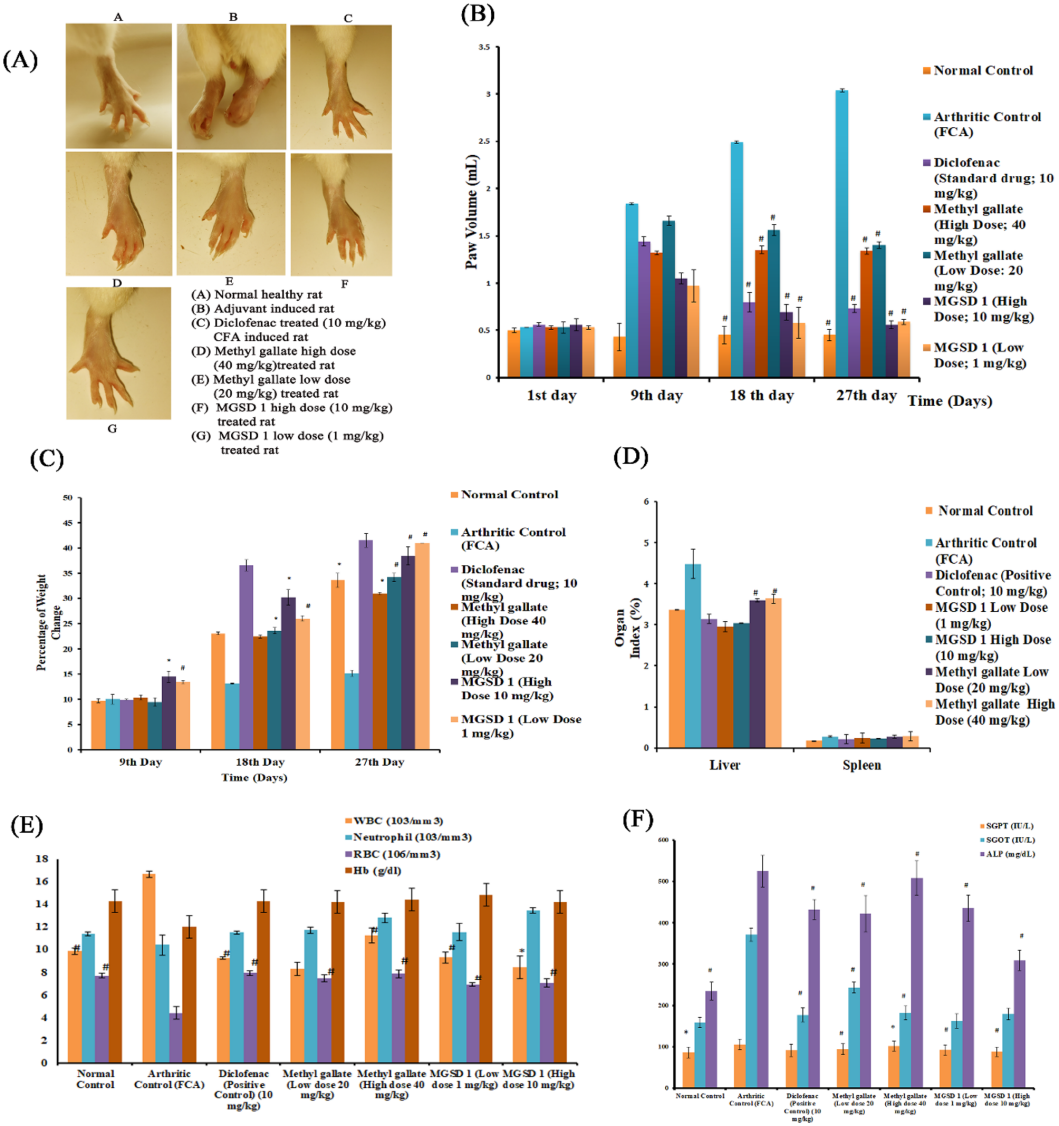
Effect of methyl gallate and MGSD 1 on hind paw volume (**A**,**B**), change in body weight (**C**), organ index (**D**), haematological parameters (**E**) and biochemical parameters (**F**) after FCA induction. Values are expressed as mean ± SEM (n = 6). Symbols represent statistical significance ^#,^*. ^#^p < 0.01 and ^*^p < 0.05 as compared to arthritic control.

Paw volume was found to be significantly increased in the arthritic control group compared to the healthy control group throughout the experiment. The left hind paw that received the intraplantar injection of FCA, experienced normal inflammatory swelling and redness from day one till day nine. In the arthritis control group, the paw swelling persisted throughout the trial, and by the 28th, the injected limb had a gross deformity. The paw volume was measured at the initial day of FCA induction and later the paw volume was measured at successive intervals of 9 days up to 28th day. Initially, inflammation was acute and reached a chronic state by the 9th day and the severity of chronic inflammation was seen as increased paw volume. The drug treatment started on the 9th day and lasted up to 28th day and volume was measured. The increased paw oedema was decreased by the treatment with methyl gallate (40 mg.kg^–1^) and MGSD 1 at 10 mg.kg^–1^ following 12–28th day after FCA administration (p < 0.01 and p < 0.05). The results are shown as the Table 4. Treatment with methyl gallate showed a decrease in paw volume but the derivative showed a significant reduction in the paw volume compared to standard drug diclofenac, even at one-tenth of the strength of the administered standard at 10 mg. kg^–1^. The inhibition of oedema formation was calculated, and diclofenac showed 59% while MGSD 1 showed 60–70% at 10 mg. kg^–1^ and the methyl gallate with an inhibition of 35–45% at 40 mg.kg^-1^ (Fig. 5 and Table 4). This analysis shows that the derivative, MGSD 1 is effective at a magnificently low strength compared to the standard, diclofenac, numerically amounting to more than ten-fold effectiveness.

**Table 4:** Paw volume measurement after treatment with methyl gallate and its synthetic derivative.

Group	Paw volume (mL) (mean ± SEM)				Percentage of inhibition
	Initial day	9th day	18th day	28th day	
Healthy rat	0.50 ± 0.05	0.53 ± 0.16^#^	0.55 ± 0.22^#^	0.53 ± 0.08^#^	
Arthritic control	0.53 ± 0.05	1.84 ± 0.16	2.49 ± 0.05	3.04 ± 0.05	
Diclofenac (10 mg.kg^−1^)	0.56 ± 0.05	1.44 ± 0.16	0.8 ± 0.22^#^	0.73 ± 0.08^#^	59%
Methyl gallate (20 mg.kg^−1^)	0.56 ± 0.05	1.32 ± 0.16*	1.24 ± 0.22^#^	1.18 ± 0.08^#^	45%
Methyl gallate (40 mg.kg^−1^)	0.50 ± 0.05	1.66 ± 0.18*	1.56 ± 0.24*	1.4 ± 0.09^#^	37%
MGSD 1 (1 mg.kg^−1^)	0.50 ± 05	0.97 ± 0.16	0.68 ± 0.22*	0.59 ± 0.08^#^	68%
MGSD 1 (10 mg.kg^−1^)	0.50 ± 0.05	1.05 ± 0.16	0.69 ± 0.22^#^	0.56 ± 0.08^#^	70%

Values are expressed as mean ± SEM (n = 6). Symbols represent statistical significance ^#^,*. ^#^p < 0.01 and *p < 0.05 as compared to arthritic control.

#### Body weight measurement

Bodyweight also showed variations at the experiments. In the FCA treated group, there was a gradual decrease in the body weight after the 9th day, while increase in body weight was observed in the drug-treated and positive control groups. There was a marked decrease in body weight seen in the RA condition, related to tissue destruction owing to the lysis of muscle proteins by lysosomal protease-mediated by prostaglandin E_2_ (PGE_2_) and a decreased absorption of glucose and leucine in rat's intestine, demonstrated by ^14^C–glucose and ^14^C–leucine analysis^27^. In addition to this, most of the autoimmune disorders resulted in a decrease in body weight due to increased production of inflammatory cytokine, loss of appetite and increased energy expenditure^2,28^.

The bodyweight of each group was monitored every day and results of successive interval of 9 days were tabulated. It was found that normal rats at the end of the experiment showed a slight increase in body weight, while adjuvant-induced groups showed an increase in body weight up to 9th day and a lesser rate of growth at last days of the experiment (Fig. 5 and Table 5). Rats treated with diclofenac showed an increase in body weight gain, though statistically not significant. Treatment with methyl gallate and MGSD 1 showed increase (p < 0.01 and p < 0.05) in body weight gain, compared to the arthritic group which showed statistically significant increase.

**Table 5:** Change in body weight after the treatment with methyl gallate and its synthetic derivative.

Group	Percentage of body weight (Mean ± SEM)		
	9th day	18th day	28th day
Healthy rat	9.10 ± 0.40	22.56 ± 0.25	32.29 ± 1.41*
Arthritic control	8.85 ± 1.67	14.67 ± 0.79	18.30 ± 0.31
Diclofenac (10 mg.kg^−1^)	9.9 ± 0.07	36.55 ± 1.10	41.52 ± 1.36
Methyl gallate (20 mg.kg^−1^)	9.43 ± 0.84	23.61 ± 0.65	34.17 ± 0.23*
Methyl gallate (40 mg.kg^−1^)	10.34 ± 0.52	22.42 ± 0.28	30.99 ± 0.23^#^
MGSD 1 (1 mg.kg^−1^)	13.40 ± 0.26^#^	25.99 ± 51^#^	40.15 ± 0.00^#^
MGSD 1 (10 mg.kg^−1^)	14.47 ± 1.13*	30.20 ± 1.57^#^	38.44 ± 1.81^#^

Values are expressed as mean ± SEM (n = 6). Symbols represent statistical significance ^#^,*. ^#^p < 0.01 and *p < 0.05 as compared to arthritic control.

#### Organ index

The spleen and liver indices were analyzed after the final day of experiment. Spleen and liver indices increased in the FCA induced group where the enlargement in spleen and liver indicated the chronic state of RA. In the arthritic state, there was a marked decrease in body weight resulting in splenomegaly and hepatomegaly. Both these conditions occur as a result of profound induction of extramedullary hematopoiesis in the red pulp in conjunction with pyogranulomatous inflammation in the red pulp and capsule. Hepatomegaly occurs as a result of hypertrophy of hepatocytes and might be beneficially affected by the treatment. FCA induced arthritic group showed a significant increase in the liver index when compared to normal rats. Compared to the control, the arthritic group showed significant splenomegaly. The standard drug Diclofenac visibly reverses this condition. Methyl gallate and MGSD 1 significantly do not reverse this condition, at the applied dose. This could suggest that both the doses of methyl gallate (20 and 40 mg.kg^–1^) and 1 mg.kg^–1^concentration of MGSD 1 are unable to cause changes. Higher concentration of MGSD 1, 10 mg.kg^–1^ is able to decrease splenomegaly (almost comparable to the diclofenac treated groups even though it is statistically insignificant) (Fig. 5 and Table 6).

**Table 6:** Effect of methyl gallate and its derivative on Organ index after induction of FCA.

Group	Organ index (%)	
	Liver	Spleen
Healthy rat	3.16 ± 0.14	0.17 ± 0.00
Arthritic control	4.48 ± 0.16	0.28 ± 0.03
Diclofenac (10 mg.kg^−1^)	3.18 ± 0.11	0.21 ± 0.01
Methyl gallate (20 mg.kg^−1^)	3.59 ± 0.03^#^	0.27 ± 0.01
Methyl gallate (40 mg.kg^−1^)	3.63 ± 0.11^#^	0.28 ± 0.02
MGSD 1 (1 mg.kg^−1^)	3.21 ± 0.12	0.24 ± 0.00
MGSD 1 (10 mg.kg^−1^)	3.18 ± 0.48	0.22 ± 0.00

Values are expressed as mean ± SEM (n = 6). Symbols represent statistical significance ^#^. ^#^p < 0.01 as compared to arthritic control.

#### Hematological and biochemical parameters

Blood parameters like Hb, RBC, WBC, lymphocytes and neutrophils were analyzed for all the treated and untreated groups of animals and the results are shown in Fig. 5 and Table 7. The experimental results showed that the parameters were tending to attain normalcy in the drug-treated group. All the evaluated hematological parameters of the FCA-control group showed an extraordinary range of results. Mainly WBC, neutrophil, and lymphocyte count were increased, and RBC and haemoglobin content was decreased in the arthritic rat. The anomalous behavior in the RBC, hemoglobin level in FCA control group due to the development of anemic condition, a clinical manifestation of RA^8,29–31^. Hemoglobin and RBC showed decrease and WBC count, lymphocyte count, and neutrophil count showed increase during the FCA induction. Among the treated groups, these parameters attained normalcy. Methyl gallate and MGSD 1 administration at higher dose showed better effect (p < 0.01 and p < 0.05) compared to the FCA-control. Results showed that the hematological parameters became normal during the treatment with both the compounds (Fig. 5 and Table 7).

**Table 7:** Haematological [*WBC* White blood cells, *RBC* red blood cells, *Hb* Haemoglobin] and biochemical parameter [*SGPT* serum glutamic pyruvic transaminase, *SGOT* Serum glutamic-oxaloacetic transaminase, and *ALP* alkaline phosphatase] observed during FCA-induction and after the treatment with methyl gallate and its synthetic derivative.

Group	WBC (10^3^/mm^3^)	Neutro phil (10^3^/mm^3^)	RBC (10^6^/mm^3^)	Hb (g/dl)	Lymphocyte (10^3^/mm^3^)	SGPT (IU/L)	SGOT (IU/L)	ALP (mg/dL)
Healthy rat	9.85 ± 0.3^#^	11.4 ± 0.15	7.71 ± 0.22^#^	14.3 ± 0.3	71.3 ± 0.40	74 ± 1.45*	176 ± 6.81^#^	370 ± 10.33^#^
Arthritic control	22.50 ± 1.76	21.46 ± 0.14	5.00 ± 0.55	12 ± 0.65	83.9 ± 1.94	106 ± 2.02	371 ± 6.06	525 ± 48.04
Diclofenac (10 mg.kg^−1^)	9.25 ± 0.10^#^	11.5 ± 0.15	7.92 ± 0.18^#^	14.3 ± 0.4	73.2 ± 1.64	91 ± 1.20 ^#^	210 ± 1.73^#^	432 ± 34.25^#^
Methyl gallate (20 mg.kg^−1^)	8.3 ± 0.6	11.7 ± 0.25	7.47 ± 0.32^#^	14.2 ± 0.65	79 ± 0.79	94 ± 3.17^#^	243 ± 13.28^#^	422 ± 41.60^#^
Methyl gallate (40 mg.kg^−1^)	11.25 ± 0.62^#^	12.8 ± 0.40	7.87 ± 0.34^#^	14.4 ± 0.74	78.6 ± 1.85	102 ± 2.18^#^	183 ± 6.38^#^	509 ± 31.54^#^
MGSD 1 (1 mg.kg^−1^)	9.3 ± 0.48^#^	11.55 ± 0.76	6.93 ± 0.18^#^	14.8 ± 0.42	75.8 ± 0.21	85 ± 2.60	162 ± 7.57^#^	435 ± 72.16^#^
MGSD 1 (10 mg.kg^−1^)	8.45 ± 0.98*	13.45 ± 0.23	7.075 ± 0.37^#^	14.2 ± 0.26	75.9 ± 3.44	82 ± 1.73	179 ± 3.52^#^	309 ± 54.42^#^

Values are expressed as mean ± SEM (n = 6). Symbols represent statistical significance ^#^,*. ^#^p < 0.01 and *p < 0.05 as compared to arthritic control.

Liver function markers are the indicators of chronic hepatic injury and serum glutamic pyruvic transaminase (SGPT), serum glutamic-oxaloacetic transaminase (SGOT) in serum, have a high impact on the formation of inflammatory mediators like bradykinin. A direct relationship between the enhanced activity of serum alkaline phosphatase (ALP) and chronic inflammation is also observed^31^. In our study it was seen that injection of FCA increased the level of SGPT, SGOT and alkaline phosphatase (ALP). Methyl gallate (40 mg.kg^–1^), MGSD 1 (10 mg.kg^–1^) and diclofenac (10 mg.kg^–1^) (p < 0.05) treatment showed a significant decrease in these enzyme levels tending towards normalcy. Thus, methyl gallate and MGSD 1 have the ability to control the elevated levels of liver enzymes in arthritis (Fig. 5 and Table 7) and also did not show any liver damage at tested doses of compounds.

#### Histopathology

**Figure 6:**
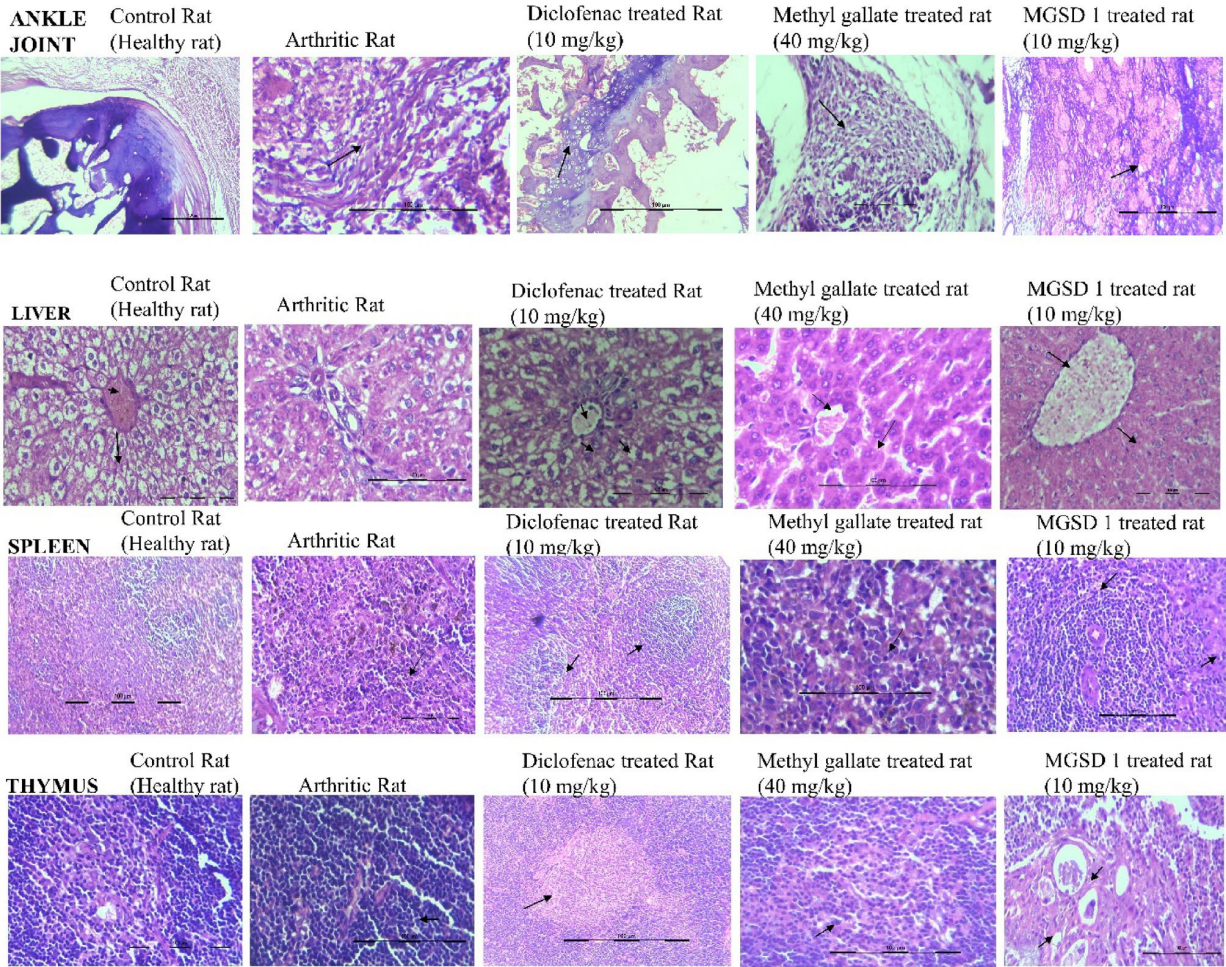
Histology of ankle joints, liver, spleen, and thymus.

Histopathological evaluation of the ankle joints, liver and spleen was performed to identify the level of inflammation and changes in the tissues at the end of the experiment. The photographs of the tissue sections are presented below (Fig. 6).

##### Histology of ankle joints

Normal control rats exhibited typical joint physiology and FCA induced rats showed extensive neutrophil infiltration in the synovium, leading to oedema and increased vascularity and cartilage destruction (Fig. 6). Methyl gallate administration (40 mg.kg^-1^) showed synovial space with neutrophil infiltration, although much less than that of arthritic control. Whereas MGSD 1 (10 mg.kg^-1^) treatment reduced oedema formation and neutrophil infiltration was found very low. In diclofenac treated rats the synovial joint was seen free of infiltration and oedema. Taken together, the results of histopathological evaluation suggested that MGSD 1 was more effective than diclofenac and methyl gallate to attenuate inflammation of FCA induced rats.

##### Histology of liver

Normal array of hepatocytes and central sinusoid area were visible in the liver of healthy rats whereas in FCA induced rats, normal array of cells was disrupted, and necrotic cells were visible. After treating with diclofenac or methyl gallate, hepatocyte arrays and central sinusoid area were seen to regain normalcy and a few necrotic cells were still visible. The MGSD 1 treatment showed higher efficacy in reverting tissues to the normal condition compared to methyl gallate and diclofenac, showing central sinusoid visible and hepatic array of cells normal (Fig. 6).

##### Histology of spleen

Spleen consists of the major functional zones, white pulp and red pulp. In the normal healthy rat, the red and white pulp area could be distinguishable, and highly proliferating germinal centers were also visible. The white pulp is found around the central arteriole and is made up of a periarteriolar lymphoid sheath (PALS). FCA induced rats had a highly proliferating spleen and the functional zone of the spleen was indistinguishable from the germinal center. While treatment with diclofenac, the periarteriolar lymphoid sheath regained normalcy as compared to arthritic control and also found that the germinal center is proliferating. Treatment with methyl gallate showed a lesser degree of spleen reversion and white pulp and red pulp area were indistinguishable due to high rate of proliferation, whereas MGSD 1 treatment seemed to be more efficient in controlling the inflammatory response than methyl gallate and diclofenac. Germinal centers were still proliferative whereas white pulp areas showed limited proliferation of cells (Fig. 6).

##### Histology of thymus

A normal histology of the thymus gland showed proliferating lymphocytes in the outer cortex and lighter stained medullary region with mature lymphocytes and Hassall's corpuscles. On the other hand, the arthritic control rat showed a highly proliferative thymus gland and several thymocytes high in both the outer and inner zone of the germinal center. Other epithelial cells were indistinguishable. Diclofenac treatment was followed by a reduction in the number of neutrophils. Rats treated with a high dose of methyl gallate seemed to be effective in controlling inflammation-based T-Cell proliferation. The outer area of the germinal center has a high density of thymocytes indicating proliferation and medullary region consisting of a few lymphocytes and increased Hassall's corpuscle. Similarly, the MGSD 1 was more effective than the parent compound in reverting inflammation-based T-Cell proliferation. The cortical region has normal lymphocytes and epithelial cells, and the medullary region consists of a few lymphocytes and increased Hassall's corpuscles (Fig. 6).

#### Real-time experiment

This study aimed to gain insight into the mechanism by which methyl gallate and its derivative influence the chronic inflammatory condition in the rat model. In the present study, the inflammatory pathway genes were identified, and their relative expression was quantified (*COX-2* and *TNF α*). RNA was isolated from the hind paw tissue of rats and its concentration was analyzed through Qubit fluorometer. RNA was isolated by Trizol method and agarose gel electrophoresis performed. After the isolation protocol, complementary DNA was synthesized and followed the quantitative PCR with standard conditions. Primers of the targets were designed in Primer3Plus and primer validation was carried out. PCR annealing temperatures were standardized to amplify the specific genes. All the primer pairs were highly specific and resolved into single bands corresponding to amplification products of expected size on 2% agarose gel electrophoresis. Primer specificity was further confirmed by a single distinct peak on the melt curve. The specificity of primer pairs ensures the sequence quality of respective gene templates. Among the three endogenous reference genes analyzed, Cq values obtained from the amplification curve of the real-time PCR for *GAPDH* were found to be more stable than *HPRt-1* and *β-Actin* in normal control and treated samples. Hence *GAPDH* was used as the internal control for the gene expression analyses.

**Figure 7:**
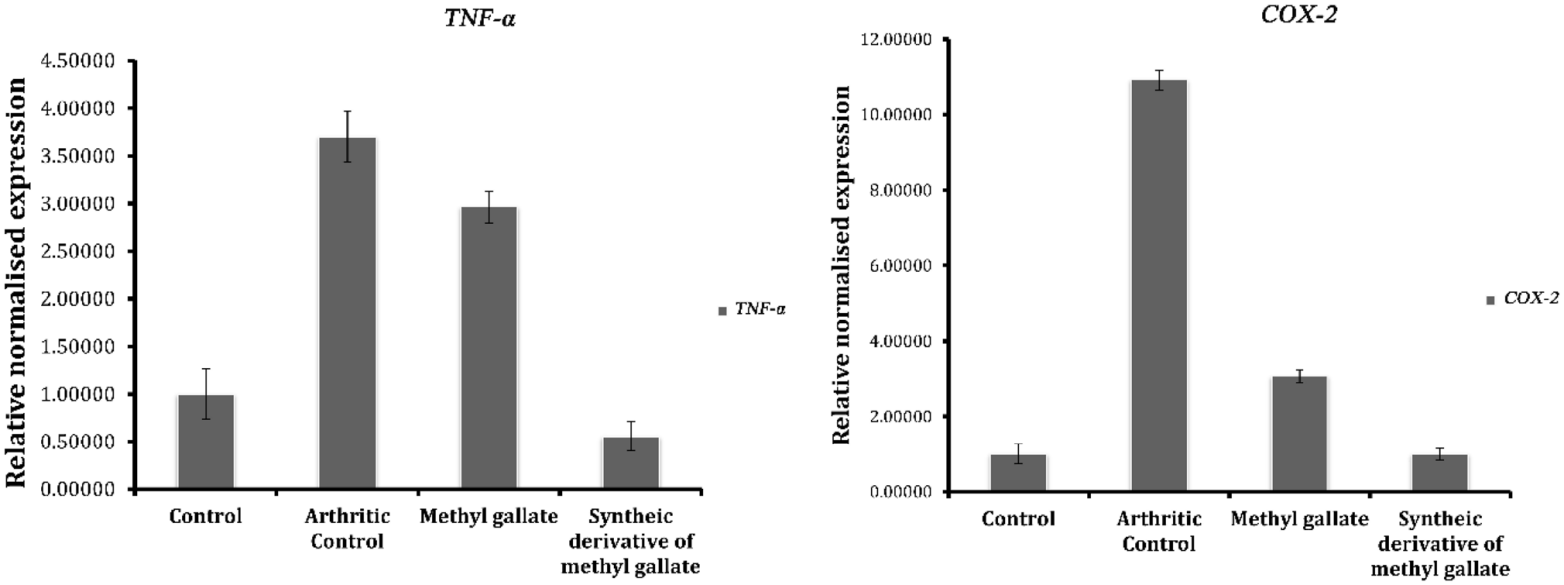
Relative changes of *TNF-α* and *COX-2* expression normalized to *GAPDH* in the normal control, arthritic control and drug-treated groups.

In the next step, real-time experiment was carried out for quantifying the genes involved in the inflammatory pathway of arachidonic acid metabolism of FCA induced groups, FCA induced diclofenac treated groups and for FCA induced drug-treated groups. The relative expression of the *COX-2* and *TNF-α* are shown in Fig. 7. In the present study, the pro-inflammatory cytokine was up-regulated in the arthritic control rat and the histology of tissues showed bone erosion, synovial hyperplasia and cartilage damage. One of the reasons behind increased pro-inflammatory cytokine was the activation of the NF-κB pathway activated by a proteasomal breakdown of I-kappa B (I-κB) during the chronic inflammatory state. Hence, the production of pro-and anti-inflammatory mediators was highly correlated with gene expression through the NF-κB pathway. It is clear that the up-regulated *COX-2* and *TNF-α* gene expressions were suppressed during the treatment with methyl gallate and synthetic derivative. The gene expression studies showed ten-fold increase of *COX-2* in arthritic control and treatment with methyl gallate and MGSD 1 showed significant down regulation of *COX-2*. Similarly in the case of *TNF-α*, 3.5-fold increase for arthritic control was observed and down-regulation was identified in drug-treated groups (Fig. 7). It could be assumed that the treatment with methyl gallate and its derivative significantly down regulated the gene expression by hindering the signaling pathway of NF-κB either by blocking phosphorylation or stimulating the expression of I-κB, obvious from the subdued pathological alterations in ankle joints^32,33^.

## Materials & methods

### Isolation of methyl gallate

Collection of plant material complied with relevant institutional, national, and international guidelines and legislation. The plant material (the root of *B. ligulata* (wall.)) was collected from the local market, authenticated from the Botany Department, Government Brennen College, Dharmadam and voucher specimens were deposited in the Herbarium, Inter University Centre for Bioscience (No. IUCB/21/12/2016). Further plant material was shade dried and extracted with different solvent systems. For a typical plant component separation, column chromatography was done on a classic 45 cm long × 2 cm diameter glass column filled with 90 g silica. The different mesh size of silica was used for the Column (Mesh 160–200; 230–400; 400–700 and Silica gel G) that was packed using a wet packing method in hexane. The gradient elution with hexane: ethyl acetate, ethyl acetate: methanol, methanol: water in the increasing order of polarity was used in the column. The fractions were analyzed using TLC and similar fractions were combined. Purified compound was further analyzed by IR and LC MS/MS^16^.

### Designing and synthesis of MGSDs

#### Pharmacophore generation

A pharmacophore model consists of spatial arrangement of chemical features that interact with a receptor^34^. The input information for pharmacophore generation includes the 3D structures of proteins, ligands, ligand–protein complexes, active site residues etc.

#### Generation of hypothesis for e-pharmacophores (Energy optimized pharmacophores)

The 'Develop Pharmacophore hypothesis’ option in the Phase module was used for the e-pharmacophore model^35^. For this, the default pharmacophore features including hydrogen bond acceptor (A), hydrogen bond donor (D), aromatic ring (R) and hydrophobicity (H) were mapped for the imported prepared protein–ligand complex. The structure of human lipoxygenase in complex with NDGA was used for generating pharmacophore (PDB ID: 6N2W). Downloaded protein was prepared using the protein preparation wizard in the Maestro software (Maestro, v10.4, Schrodinger, LLC, NewYork, NY).

#### E-pharmacophore based virtual screening

The designed MGSDs were used for E-pharmacophore based virtual screening. The ligands were structurally optimized at near-neutral pH (7 ± 1) before screening. All plausible tautomers and stereoisomers were generated, and protonation states were assigned. The ligands were subjected to energy minimization with OPLS3 force field by the ligprep module of Maestro 10.4. To generate a subset of drugs with the desired molecular features for optimal binding to LOX-5, as mapped by the E-pharmacophore model, a pharmacophore-based virtual screening was carried out using the phase module of Schrodinger suite. The fitness scores were used to select the best hits^34,36,37^.

#### Molecular docking

The best hits obtained in the previous step were undergone further screening by molecular docking using the GLIDE module. By keeping the crystallographic ligand NDGA at the active site, a grid box was generated, around this cavity with a size of 15 × 15 × 15 Å (PDB ID: 6N2W). This space was used by ligand during the docking procedure.

#### Molecular simulation

Simulations were carried out for the best pose acquired from docking with the Desmond module of Schrodinger 10.4, utilizing TIP4P as water model^38^ and OPLS3 force field and ensemble as NPT. An orthorhombic water box was set up so that it secured the whole protein–ligand complex. To nullify the negative charge of the protein Na^+^ ions were included. The pressure and temperature were kept constant at 1 bar and 300 K. The MD simulation was performed for a time frame of 200 ns with 100 ps time steps^39^.

#### Pharmacokinetics prediction

In a drug design point of view, the pharmacokinetic properties of the designed compounds are important. Pharmacokinetic prediction was carried out with Schrödinger suite’s Qikprop module (QikProp, version 3.5, Schrödinger, LLC, New York,). Also, Qikprop can predict any possible drug lead by comparing the compound scaffolds with known databases and analyzing similarity within a class of compounds.

### Synthesis

#### Structural characterization of derivatives

^1^H NMR spectra were recorded on Bruker's AVANCE series with 400 MHz/300 MHz with CDCl_3_ as the internal standard. LC separation was performed on an LCMS Agilent1100 series using XBridge C18 5 µm 4.6 × 150 mm column and CORTECS Shield RP18 MV-Kit 50 × 3 mm 2.7 µm columns. Injection volume was 10 µL. Sample elution was done isocratically using acetonitrile and 10 mM NH_4_OAC at a flow rate of 4 mL/min and acetonitrile and 0.1% formic acid in water at a flow rate of 2 mL/min.

#### In-vitro enzyme inhibition assay

The LOX inhibition assays were performed with MGSDs. The inhibitory activity was measured by a modified spectrophotometric method^16,39^. The plant LOX pathway is in many respects equivalent to the ‘arachidonic acid cascades’ in animals. For this reason, the in vitro inhibition of LOX constitutes a good model for the screening of compounds with anti-inflammatory potential^40,41^. LOX (Lipoxidase from Glycine max (15- LOX); EC 1.13.11.12) type I-B (Soybean), and linoleic acid were purchased from Sigma (Sigma-Aldrich, UK) and used without further purification. Enzyme solution of 1.03 µM was prepared in 0.2 mM borate buffer, pH 8.5. The substrate, linoleic acid solution of 0.3 mM was also prepared in the borate buffer at the same pH. MGSD 1 soluble in water and MGSD 2 and 3 were solubilized in 1% DMSO. The final concentration of MGSDs used for the analysis was 20 µM. The assay mixture was made of 50 µL of LOX, 50 µL of the test solution and 360 µL of the substrate. The final volume was made up to 2 mL with corresponding borate buffer quantity. The activity of LOX was measured on the formation of hydroperoxy octatetraenoic acid, which was monitored at 234 nm on spectrophotometer, (HITACHI U 2900, Japan). The same procedure was repeated with triplicate for the confirmation of LOX inhibitory activity^16,39^.

#### Michaelis–Menten enzyme kinetics

To identify the mode of inhibition, enzyme kinetics assay was performed with MGSDs. For the kinetic analysis different substrate concentrations of 18, 27, 36, 45, 54, 63 µM were prepared in 0.2 mM borate buffer (H 9)^16^. The assay mixture was made of 50 µL of LOX, 50 µL of the test solution and different concentrations of the substrate. The final volume was made upto 2 mL with corresponding borate buffer quantity. The activity of LOX was measured by the formation of hydroperoxy octatetraenoic acid, which was monitored at 234 nm on spectrophotometer, (HITACHI U 2900, Japan). The reaction was measured at a time span of 300 s. The same experiment was repeated in the presence of 0.1, 0.08 and 0.06 μM concentration of MGSDs. The Line Weaver-Burk plot was constructed and Michaelis–Menten constant (K_*m*_) and Maximal Velocity (V_*max*_) were determined from the plot. From the K_*m*_ and V_*max*_ obtained, the inhibitor constant, *K*_*i*_ was calculated using the following equation derived from the Michaelis–Menten Equation:

$$K^{\prime}_{m} = K_{m} \left( {{1} + {\text{ I}}_{0} /K_{i} } \right).$$

The IC_50_was calculated using the Cheng–Prusoff equation,

$$K_{i} = {\text{IC}}_{{{5}0}} / \, \left( {{1} + \left[ {\text{S}} \right]/K_{m} } \right).$$

### Fluorescence quenching studies

The Fluoromax-4 spectrophotometer (Spectra-NanoLED source 278) was used for studying fluorescence quenching of the compound. Bandwidth set at 5 nm for both excitation and emission spectra. The wavelength was set for excitation at 280 nm and the emission was recorded at 290–450 nm using 1.0 cm cell at 296 K temperature. The LOX with a concentration of 7 μM, dissolved in borate buffer at pH 9 and ligands at the concentrations of 35, 70, 105, 140, 175, 210, 245 and 280 μM were used for the experiment^39^.

### In vivo effect of methyl gallate and MGSD 1 on chronic inflammatory conditions

#### Animals

Adult male and female Sprague–Dawley (SD) rats (130–150 g) were purchased from Kerala Veterinary & Animal Science University, College of Veterinary and Animal Sciences, Thrissur (license no. 328/PO/c/01/CPCSEA Dated 03.01.2001) and kept at controlled environment, at a constant temperature (23 ± 2 °C), humidity (60 ± 10%), and a 12/12 h light/dark cycle. Rats were acclimatized for 1 week before the experimental procedures and were allowed standard rat chow (Krish Scientists Shoppe, Bangalore) and water ad libitum. All experimental procedures were carried out following the guide for the Care and Use of Laboratory Animals. The procedures were approved by the Institutional animal ethical committee before the animal experiments begun (KULS/IAEC/2019/27).

#### Oral bioactivity studies

Acute oral toxicity of new, synthetic compounds was carried out in female Sprague Dawley rats weighing 80–100 g according to OECD guideline 420^22^. The fixed-dose level of 5, 50, 300 and 2000 mg.kg^–1^ was administered as a single dose via gavage. After the administration of drug, each animal was observed for 30 min, 1 h, 2 h, 4 h, 24 h and then 48 h up to 72 h and then everyday till 14 days period for clinical signs, gross behavior changes and mortality, if any.

#### Induction of chronic inflammation and treatment protocol

Chronic inflammation induced by Freund's Complete Adjuvant contains a suspension of heat-killed *Mycobacterium tuberculosis* in the vehicle (Chondrex, Labex Corporation). 100 μL of FCA (10 mg/mL) was injected intradermally to the left-hand paw of SD rats^42^. Hind paw volume was found to be a parameter for the measure of inflammation and analyzed within a consecutive period after chronic inflammation. Induction of inflammation became chronic by 9–12 days and then rats were divided into 7 groups having 6 rats in each group. Everything possible was done to minimize the suffering of experimental animals. Minimum number of rats only were drafted to generate reliable data. 42 rats were randomly allocated into seven groups (6 × 7).

Group I (n = 6) served as normal rats that received an equal volume of vehicle control. Group II (n = 6) was arthritic model control treated with vehicle only. Group III (n = 6) was arthritic control which received the diclofenac (10 mg.kg^–1^). Groups IV (n = 6), V (n = 6), VI (n = 6) and VII (n = 6) were treated with a low and high dose of methyl gallate (20 mg.kg^–1^, 40 mg.kg^–1^) and MGSD 1 (1 mg.kg^–1^, 10 mg.kg^–1^). The drugs were orally administered through oral gavage daily in PBS vehicle for 28 days.

#### Hind paw volume

By a plethysmometer the rat hind paw volume was measured from the initial day to final day (28th day) with successive intervals of 9 days, before and after FCA injection. The inhibition was calculated by the formula given below.

$$Percentage\,\, inhibition=1-\left(\frac{Mean \,\,changes\,\, in \,\,paw \,\,volume\,\, of\,\, treated\,\, rat}{ Mean\,\, changes\,\, in\,\, paw \,\,volume\,\, of \,\,untreated \,\,rat}\right)\times100.$$

#### Biochemical and hematological evaluation

Blood was drawn from jugular vein^43^ and collected in heparinized tubes and used for further analysis. By using Horiba 5-part hematology analyzer the blood parameters like RBC, WBC, neutrophil and hemoglobin were analyzed. Serum parameters were analyzed using Stat Fax autoanalyzer. Serum glutamic pyruvic transaminase (SGPT), serum glutamic oxaloacetic transaminase (SGOT) and alkaline phosphatase (ALP) were determined using standard diagnostic kits (Proton, Accurex).

#### Body weight measurement and organ index calculation

The bodyweight of the animals was measured every day after the commencement of the experiment till the end (28th day). The weight change was calculated using the formula:

$$\mathrm{Weight\,\, change }\left(\mathrm{\% }\right)=\frac{Wt-Wo}{Wt},$$

where *Wt* is the weight of the animal at time *t*

*Wo* is the weight of the animal on the initial day. The result was statistically compared to both normal control and FCA-control groups.

At the end of the experiments, animals were euthanized using ketamine injection. The liver and spleen were removed at the end of the experiment and weighed. The index of the organs was expressed as the percentage (%) wet weight of organ versus body weight. The organ indexes were calculated by using the following formula

$$Organ\,\, index=\left[\frac{Weight\,\, of \,\,organ\left(g\right)}{Body \,\,weight \left(g\right)}\right] \times 100.$$

#### Histological analysis

The animals used for the FCA induced arthritis were euthanized at the end of the experiment and the ankle joints were separated from the hind paw and fixed in 10% buffered formalin. Similarly, liver, spleen, and thymus were also dissected carefully and immersed in fixative, and after 24 h each specimen were decalcified in 5% nitric acid, followed by embedding in paraffin wax and 4 µm thickness sections were prepared. These prepared sections were then mounted and stained with hematoxylin and, observed under brightfield compound binocular research microscope at 100X and 400X magnification and inflammation was graded.

#### RT-PCR analyses

The relative expression of inflammatory genes was identified through quantitative RT-PCR. Initially, total RNA was isolated by using TRIzol reagent. The concentration of RNA isolated was measured in the Qubit fluorometer with the RNA HS assay kit. cDNA was synthesized by reverse transcription using total RNA (2 μg) as a template. Gene expression analysis was accomplished by real-time reverse transcription polymerase chain reaction (RT-PCR) (Biorad CFX Connect Real-Time PCR, Applied biosystems) using cDNA synthesized from the arthritic specimen. The primers for target genes were designed in Primer3 plus online tools. Designed primers were as follows: *GAPDH* (F: CATCACTGCCACCCAGAAGACTG, R: ATGCCAGTGAGCTTCCCGTTCAG); *COX-2* (F: AAAGCCTCGTCCAGATGCTA, R: ATGGTGGCTGTCTTGGTAGG); *TNF- α* (F: GCCATAGAACTGATGAGAGGGAG, R: GGTGCCTATGTCTCAGCCTCTT). The protocol described in Maxima SYBR Green/ROX qPCR master mix manual was standardized for the selected genes and followed for the experiment. Technical replicates were maintained for RT-PCR. The 20 μL RT-PCR reaction mix was prepared with 10 μL SYBR Green qPCR master mix, 2 μL of 0.625 μM each of forward and reverse primers, 1 μL of 10 ng of cDNA template and nuclease-free water (7 μL). The RT-PCR reactions were performed in Biorad CFX Connect Real-Time PCR, Applied biosystems.

### Statistical analysis

All values were expressed as mean ± SEM (*n* = 6). Statistical analysis was performed with protocol of one-way analysis of variance (ANOVA) followed by Post Hoc Tukey HSD using SPSS software version 20. A p value of < 0.05 was considered statistically significant.

### Ethics approval

This study was performed with the approval granted by the Institutional Animal Ethical Committee of Kannur University, number KULS/IAEC/2019/27, and approved by CPCSEA, New Delhi (India). The studies performed by the approval of the CPCSEA (India) conforms to the ARRIVE (https//arriveguidelines.org) guidelines and the study is reported in accordance with ARRIVE (https//arriveguidelines.org) guidelines.

## Conclusion

The present study dealt with the in silico, in vitro and in vivo anti-inflammatory properties of methyl gallate and MGSD 1. The in-silico studies proved the efficacy of carboxylic acid derivatives of methyl gallate as potent LOX inhibitors. The molecular docking result showed that MGSD 1 has a docking score of − 11.61 kcal/mol, whereas MGSD 2 and MGSD 3 have − 10.32 and − 9.02 kcal/mol, respectively. The simulation studies also proved the stable binding of MGSD 1 at the active site of LOX. The in vitro studies showed better lipoxygenase inhibitory potential for the synthetic derivatives of methyl gallate than the parent compound. The mechanism of action was studied through in vivo together with gene expression studies. RA was chosen as a model disease for chronic inflammation, and the results showed that methyl gallate and MGSD 1 reduced the symptoms of arthritis. It could be a good drug candidate to develop highly efficacious and little toxic drug for treating RA. It may be noted that the methyl gallate is required approximately five times of diclofenac for the same level of effect and the MGSD 1, is required only approximately 1/12 of diclofenac for the same level of effect as perceived from the in vivo studies with no toxic effects. The molecular mechanism behind RA was demonstrated through gene expression studies. In FCA induced arthritis several pro-inflammatory, anti-inflammatory cytokines, chemokines and lipid mediators were expressed because of inflammatory responses. Increased expression of *COX-2* and *TNF-α* were observed in the present study, whereas in treatment with compounds, there is a downregulation of genes observed. An NF-κB signaling pathway is responsible for the inducible expression of *COX-2* and *TNF-α* gene. Hence, it is concluded that the methyl gallate and its derivative may be inhibiting the NF-κB signaling pathway and results in the decreased mRNA expression of these genes to ameliorate RA.

## Supplementary Information

Supplementary Information.

## Data Availability

All data generated or analysed during this study are included in this published article [and its supplementary information files].
