# Supplementary material for: Lipoxygenase inhibitory synthetic derivatives of methyl gallate regulate gene expressions of COX-2 and cytokines to reduce animal model arthritis
Source: Sci Rep. 2023 Jun 30;13:10644. doi: 10.1038/s41598-023-37613-z (PMC10313808; doi:10.1038/s41598-023-37613-z)
Supplement: Supplementary file 1 — Supplementary Information. [file 41598_2023_37613_MOESM1_ESM.docx]

**Lipoxygenase inhibitory synthetic derivatives of methyl gallate regulate gene expressions of COX-2 and cytokines to reduce animal model arthritis** Sharanya CS^1,2^, Abhithaj J^1^, Arun KG^1^, Koti Reddy Eeda^3^, Vignesh Bhat^4^, Variyar EJ^1^,

Sabu A^1^, Haridas M^1,*^

**^1^** Department of Biotechnology and Microbiology and IUCB, Dr. Janaki Ammal Campus, Kannur University, Palayad, Thalassery, Kannur, Kerala-670661, India

**^2^**Transdisciplinary Biology, Rajiv Gandhi Centre for Biotechnology (RGCB), Thiruvanthapuram, Kerala 695014, India.

**^3^** Department of Chemistry, Vignan Foundation for Science Technology and Research, Vignan University (Deemed to be University), Vadlamudi, Guntur-522 213, India

**^4^** Department of Chemistry, Mangalore University, Mangalagangothri-574 199, Karnataka, India

- Corresponding Author: [mharidasm@rediffmail.com](mailto:mharidasm@rediffmail.com)


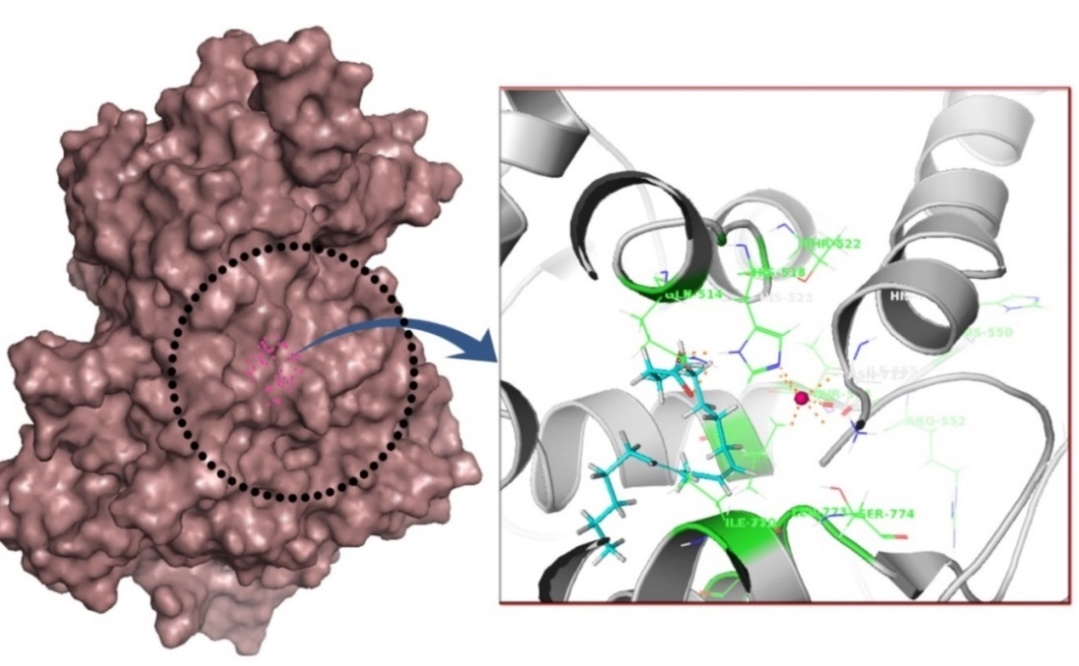


Supplementary Figure 1: Designed derivatives of methyl gallate


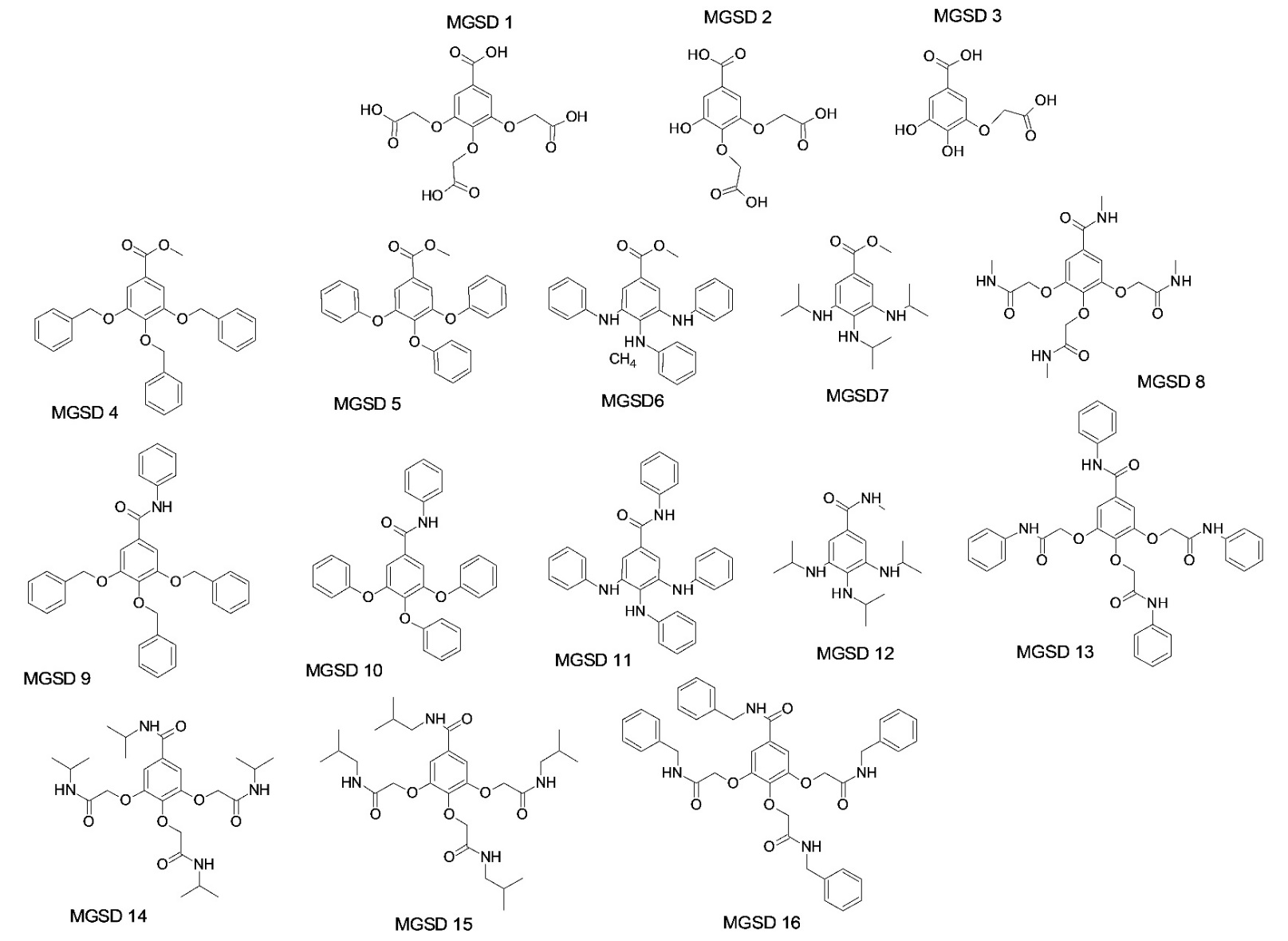


Supplementary Figure 2: The interaction of linoleic acid at the active site of lipoxygenase


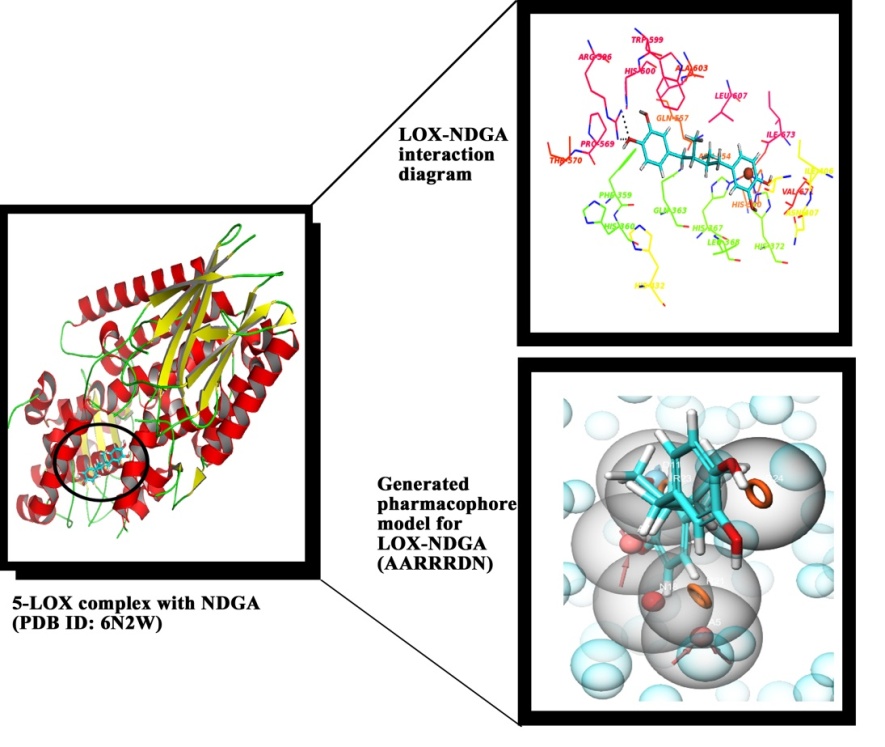


Supplementary Figure 3: Pharmacophore model of lipoxygenase generated by an e-pharmacophore method in Phase module


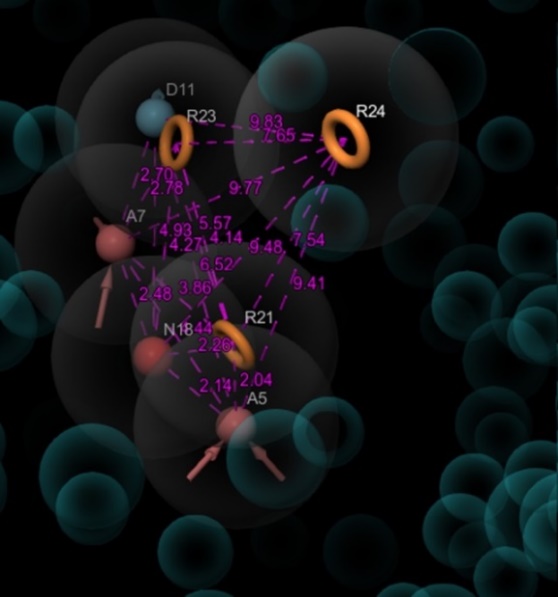


Supplementary Figure 4: The distance between each pharmacophore groups and the generated pharmacophore AARRRDN (Two hydrogen bond acceptors (A), three aromatic rings (R), the hydrogen-bond donor (D) and negative ionizable (N) group)


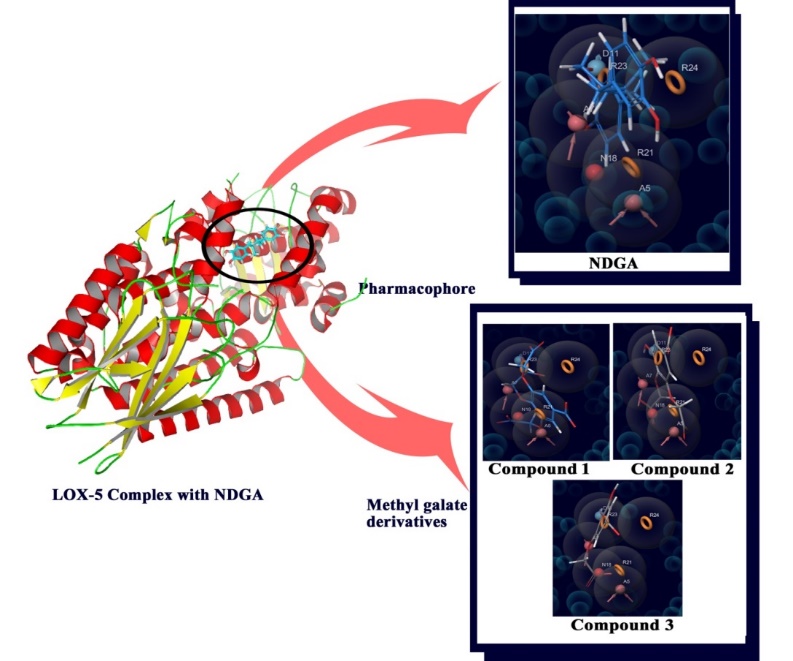


Supplementary Figure 5: Screened derivatives of methyl gallate using pharmacophore model


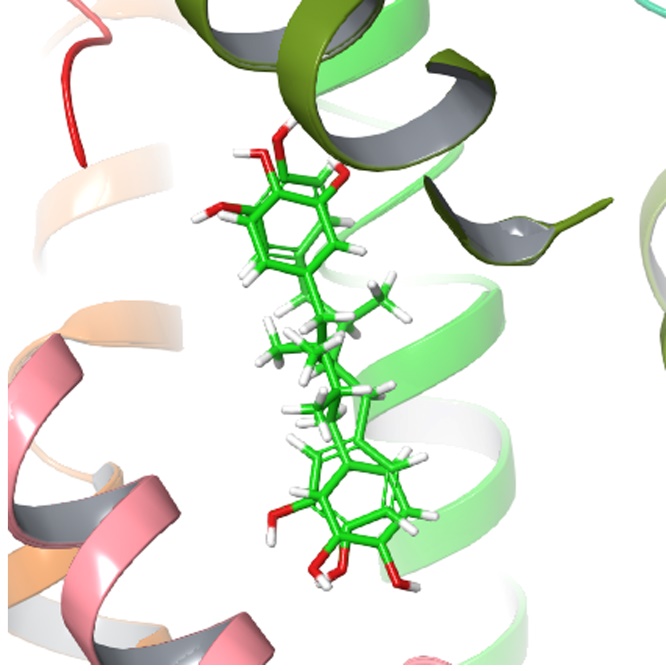


Supplementary Figure 6: Superimposed image of the docked structure of NDGA complex with lipoxygenase and crystal structure


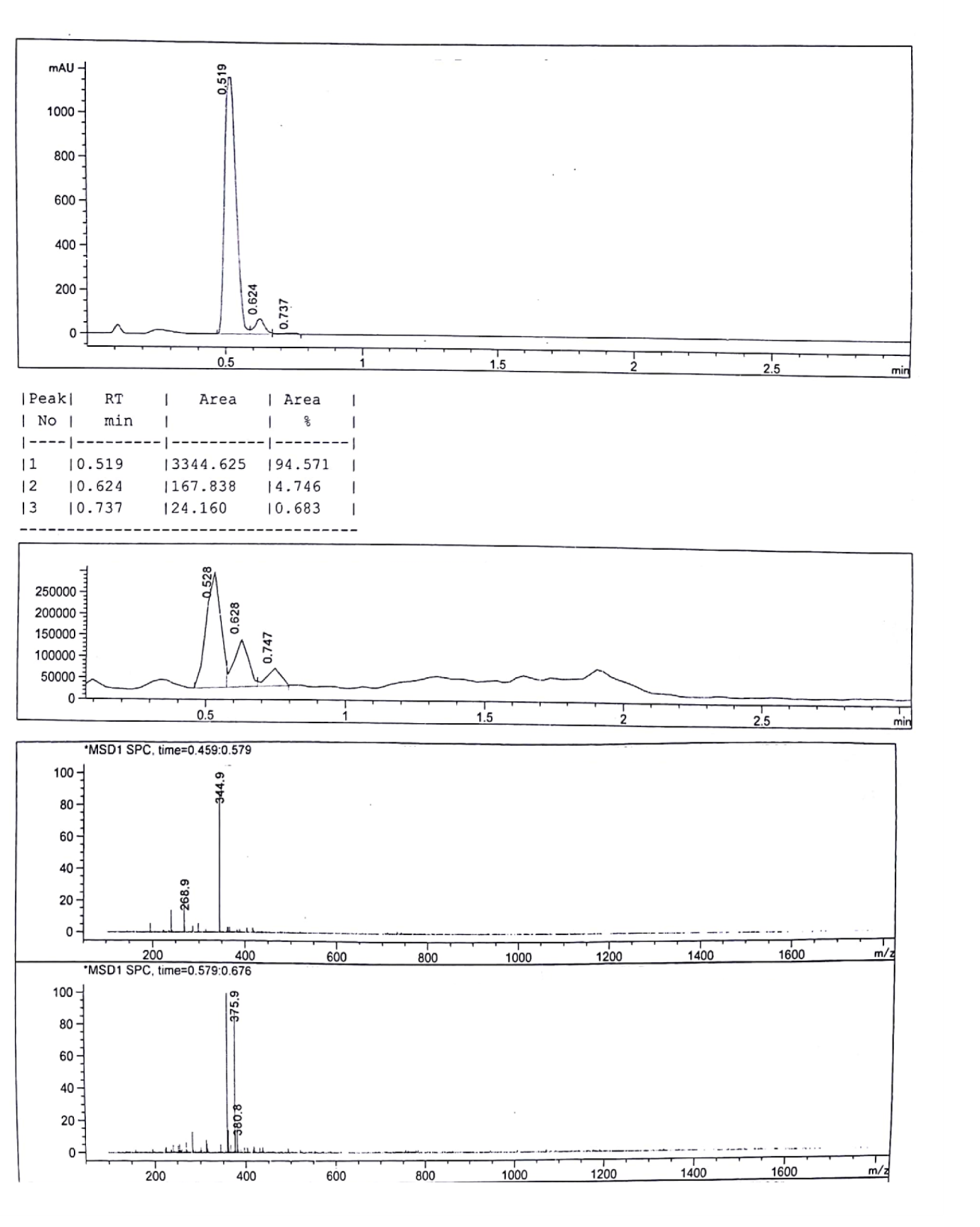


Supplementary Figure 7: LC-MS spectrum of MGSD 1 (2,2',2''-((5-carboxybenzene-1,2,3-triyl)tris(oxy)) tri acetic acid)


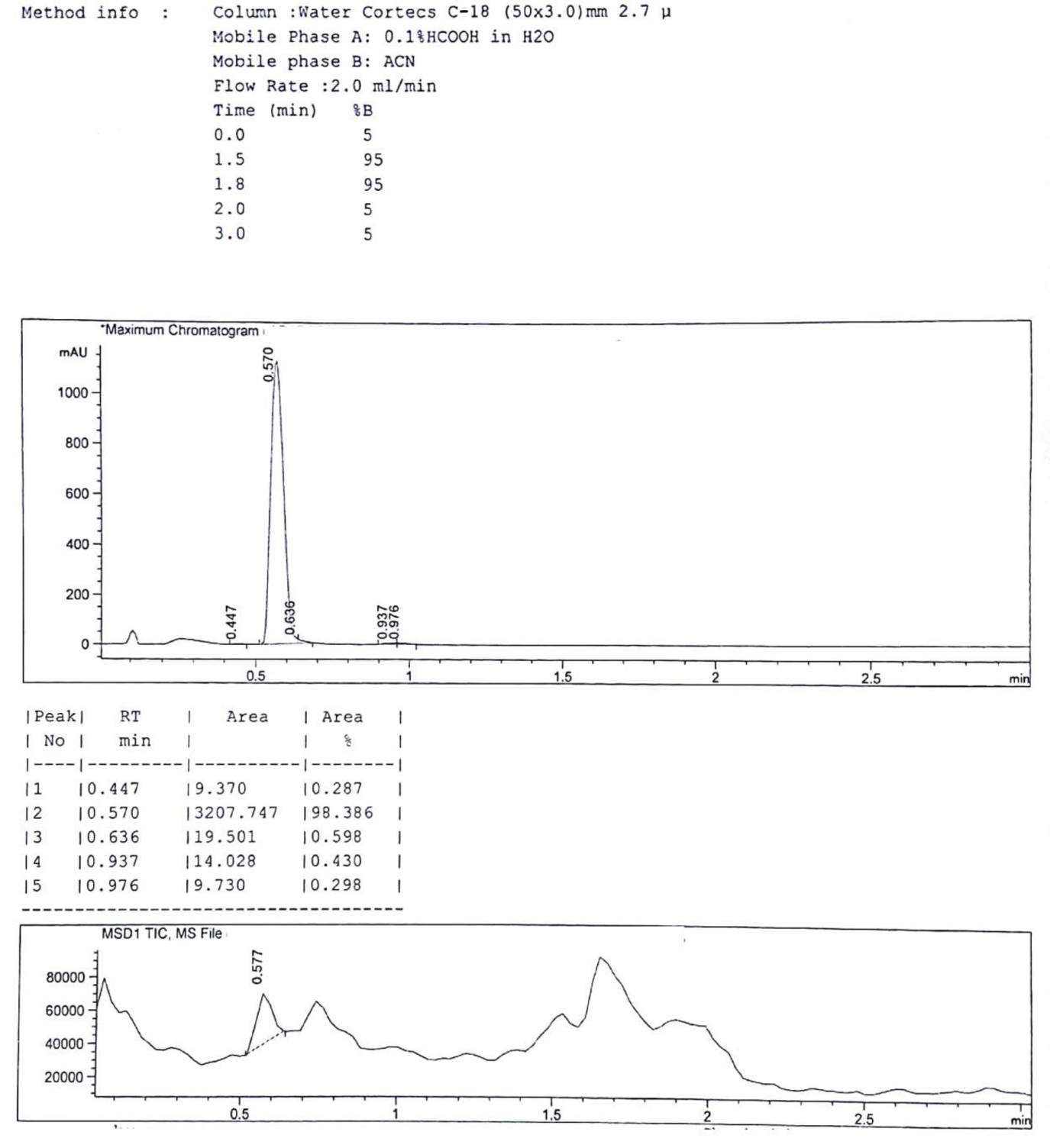


Supplementary Figure 8: LC chromatogram of MGSD 2 (2,2'-((5-carboxy-3-hydroxy-1,2-phenylene) bis(oxy)) diacetic acid)


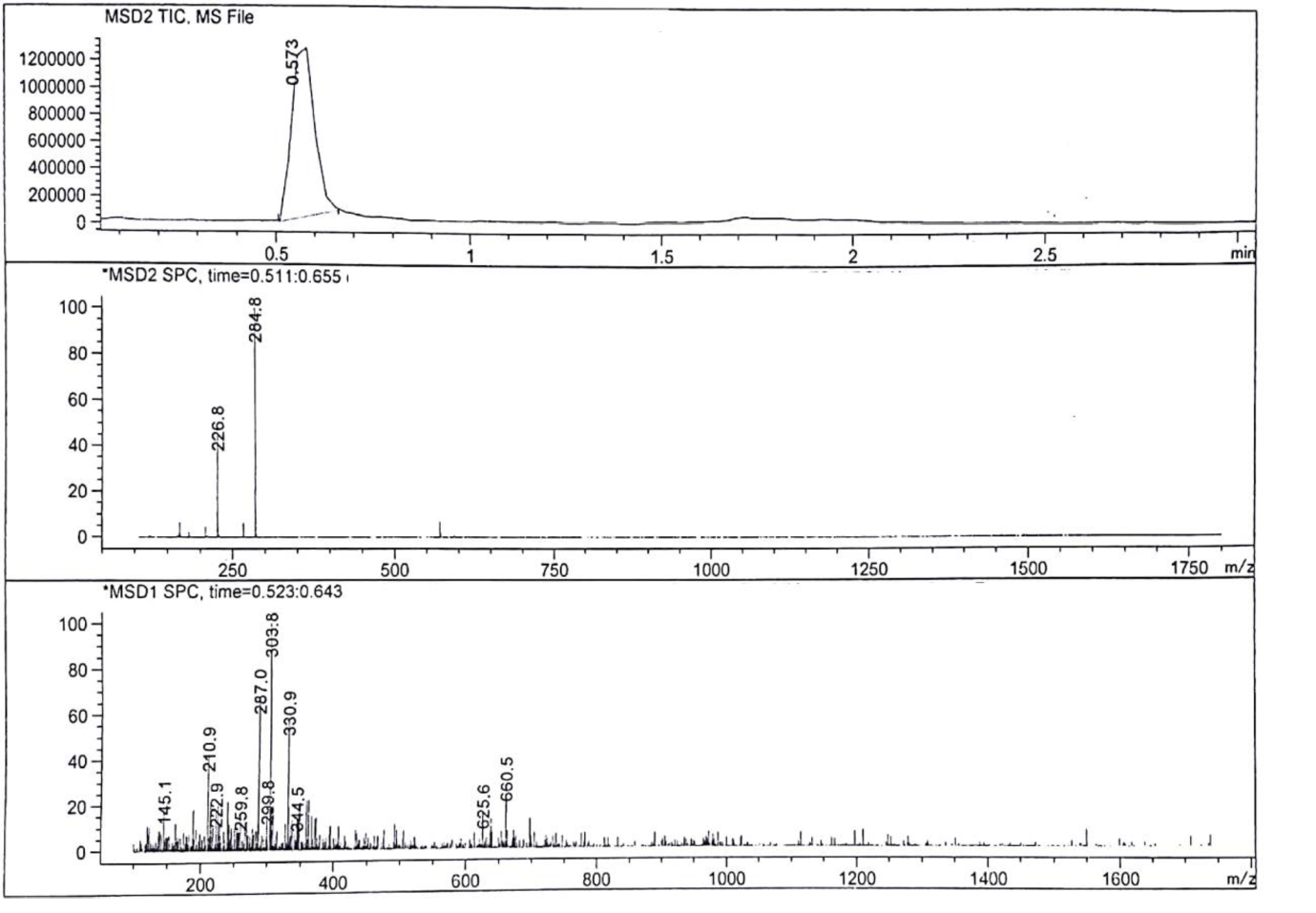


Supplementary Figure 9: LC-MS spectrum of MGSD 2 (2,2'-((5-carboxy-3-hydroxy-1,2-phenylene) bis(oxy)) diacetic acid)


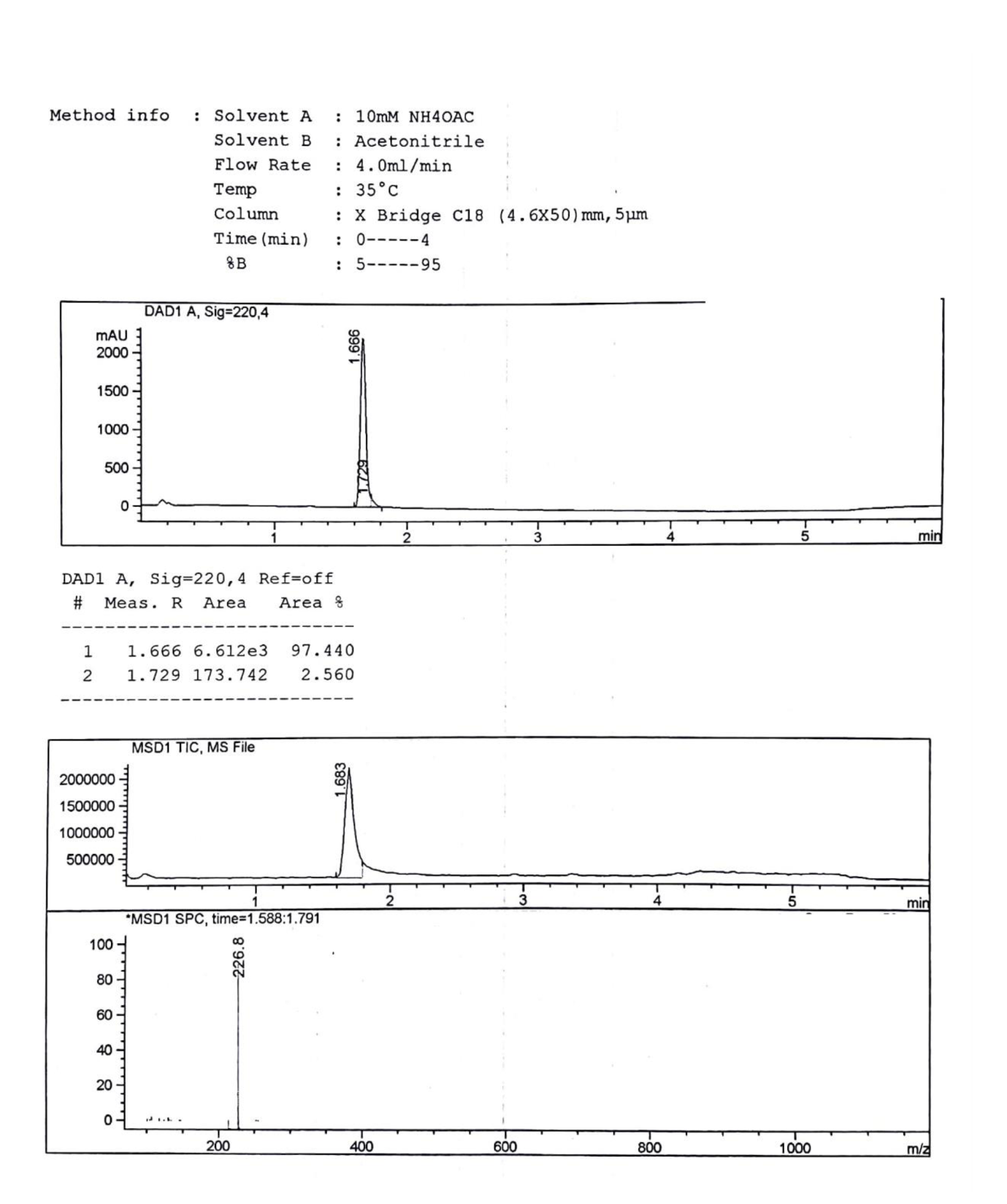


Supplementary Figure 10: LC-MS spectrum of MGSD3 (3-(carboxymethoxy)-4,5-dihydroxybenzoic acid)


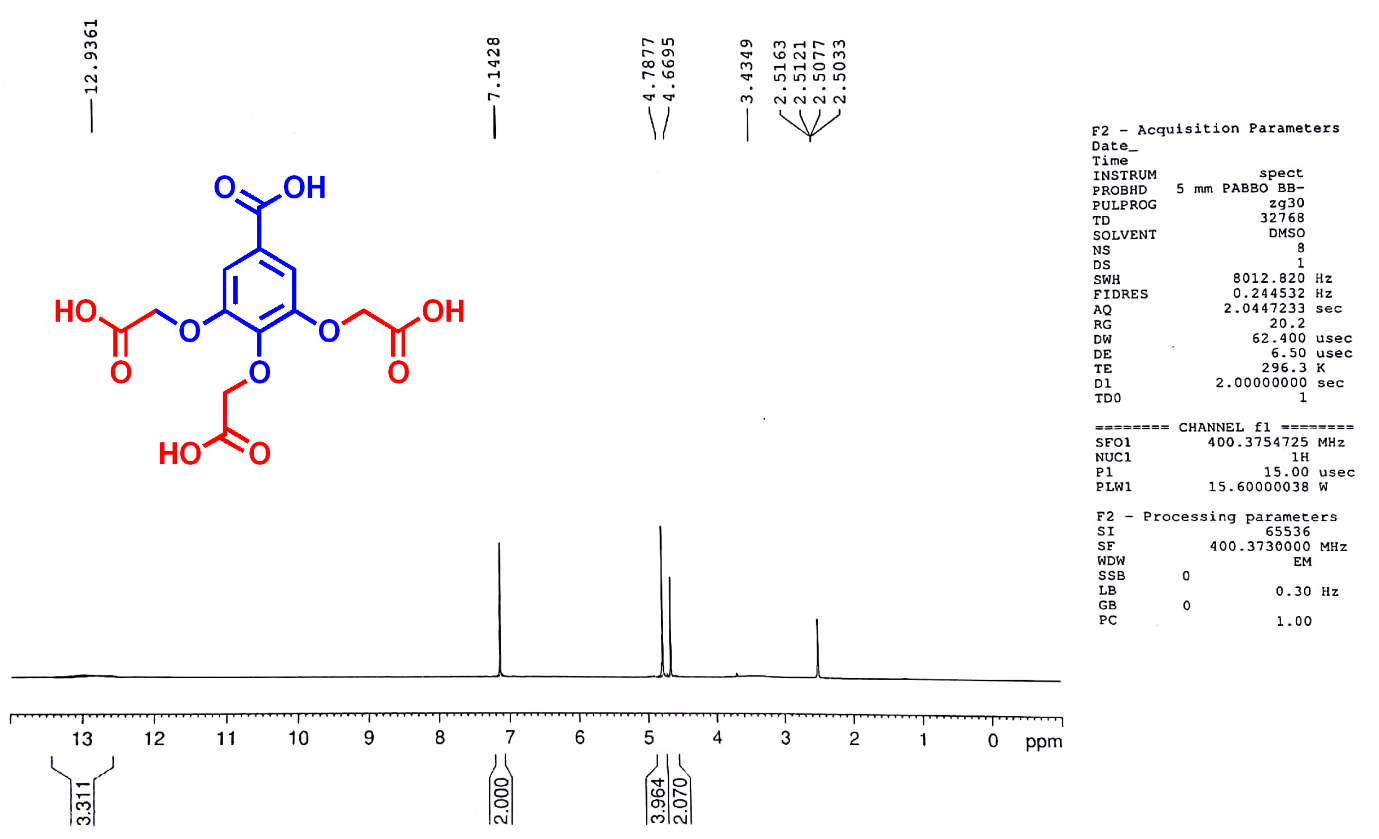


Supplementary Figure 11: ^1^H NMR of MGSD 1


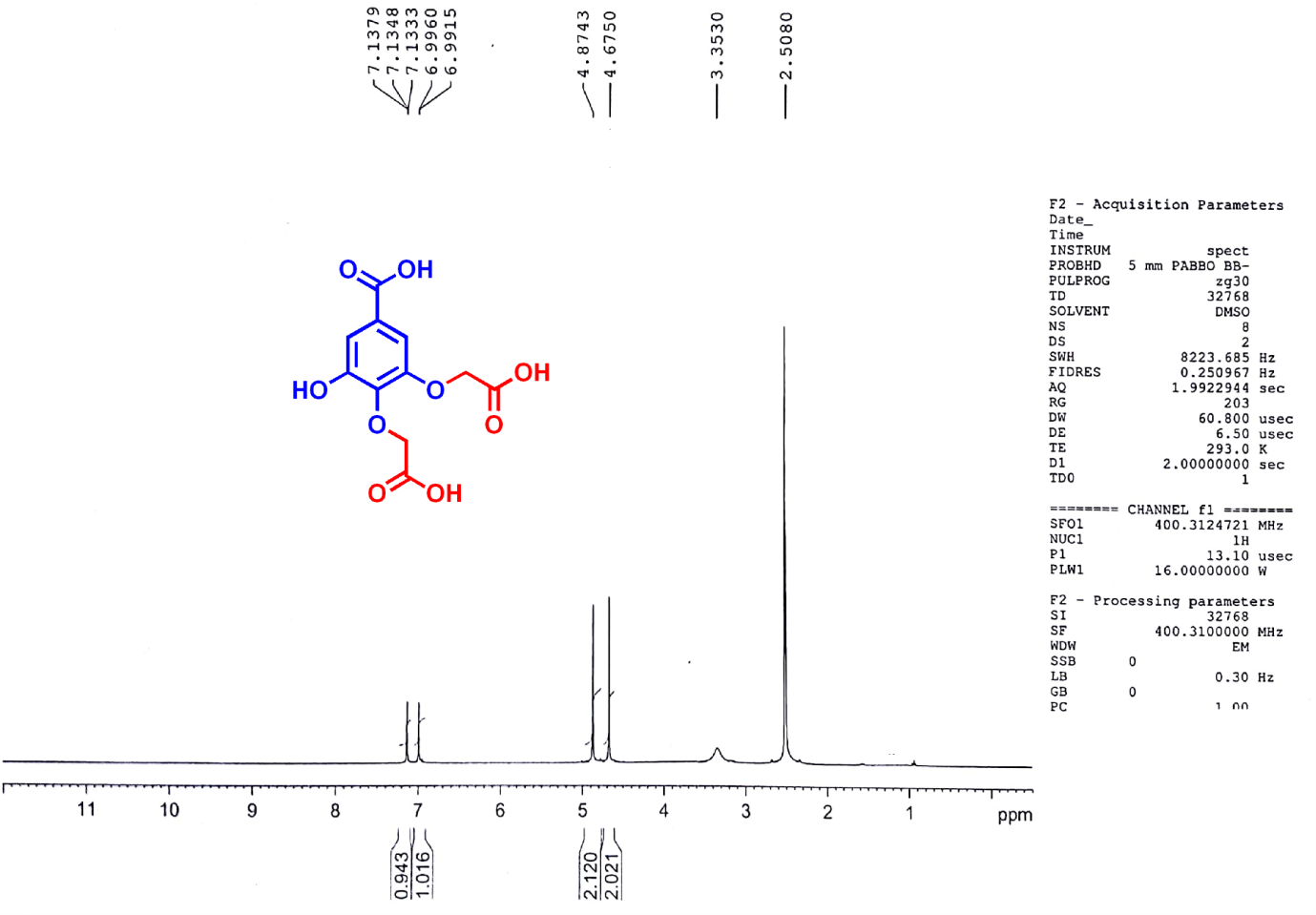


Supplementary Figure 12: ^1^H NMR of MGSD 2


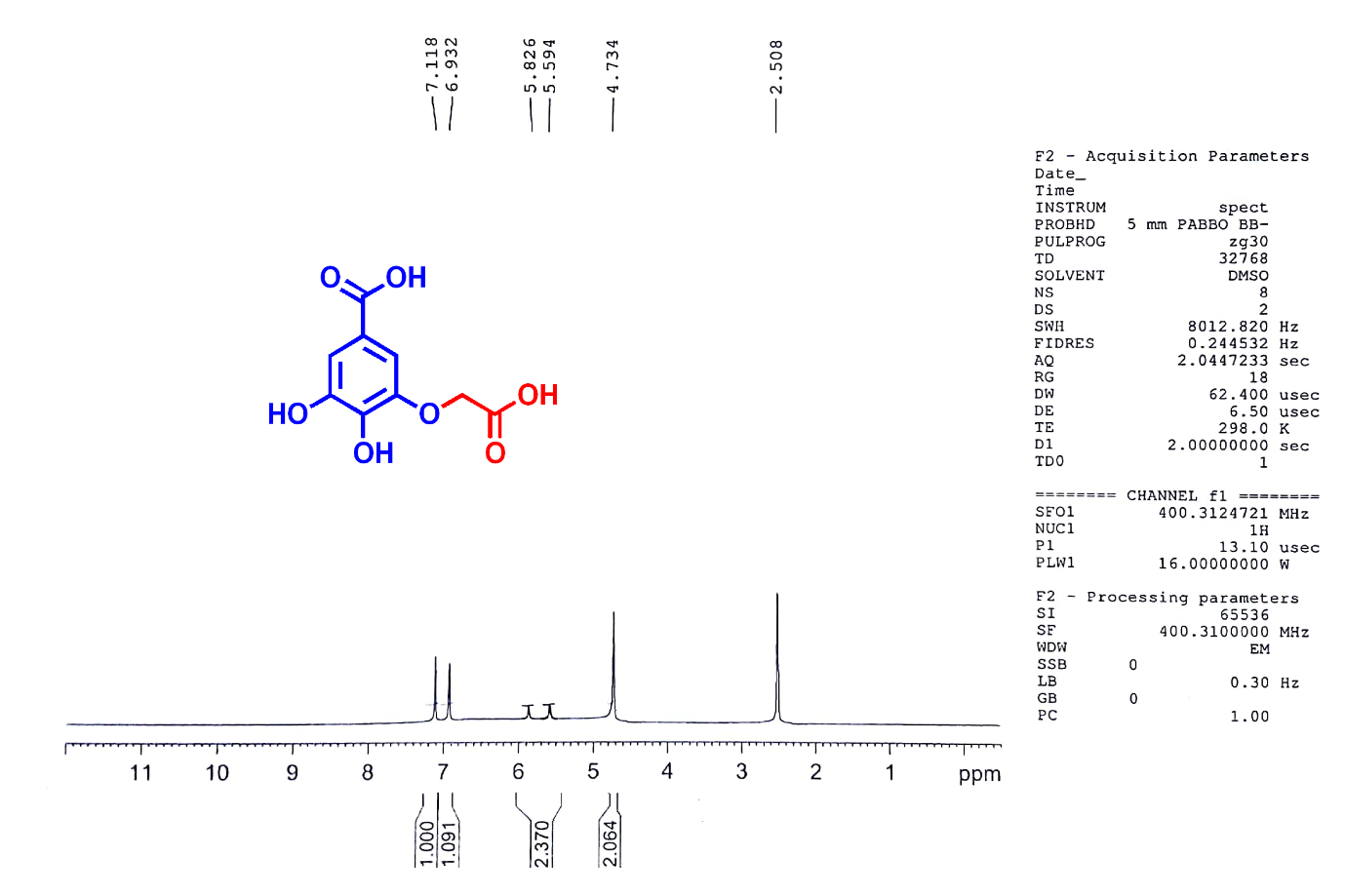


Supplementary Figure 13: ^1^H NMR of MGSD 3


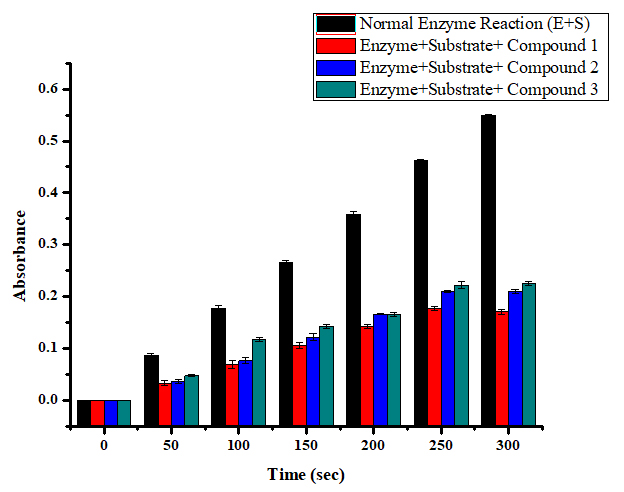


Supplementary Figure 14: Bar diagram showing lipoxygenase inhibitory profile of methyl gallate derivatives MGSD 1, MGSD 2 and MGSD 3


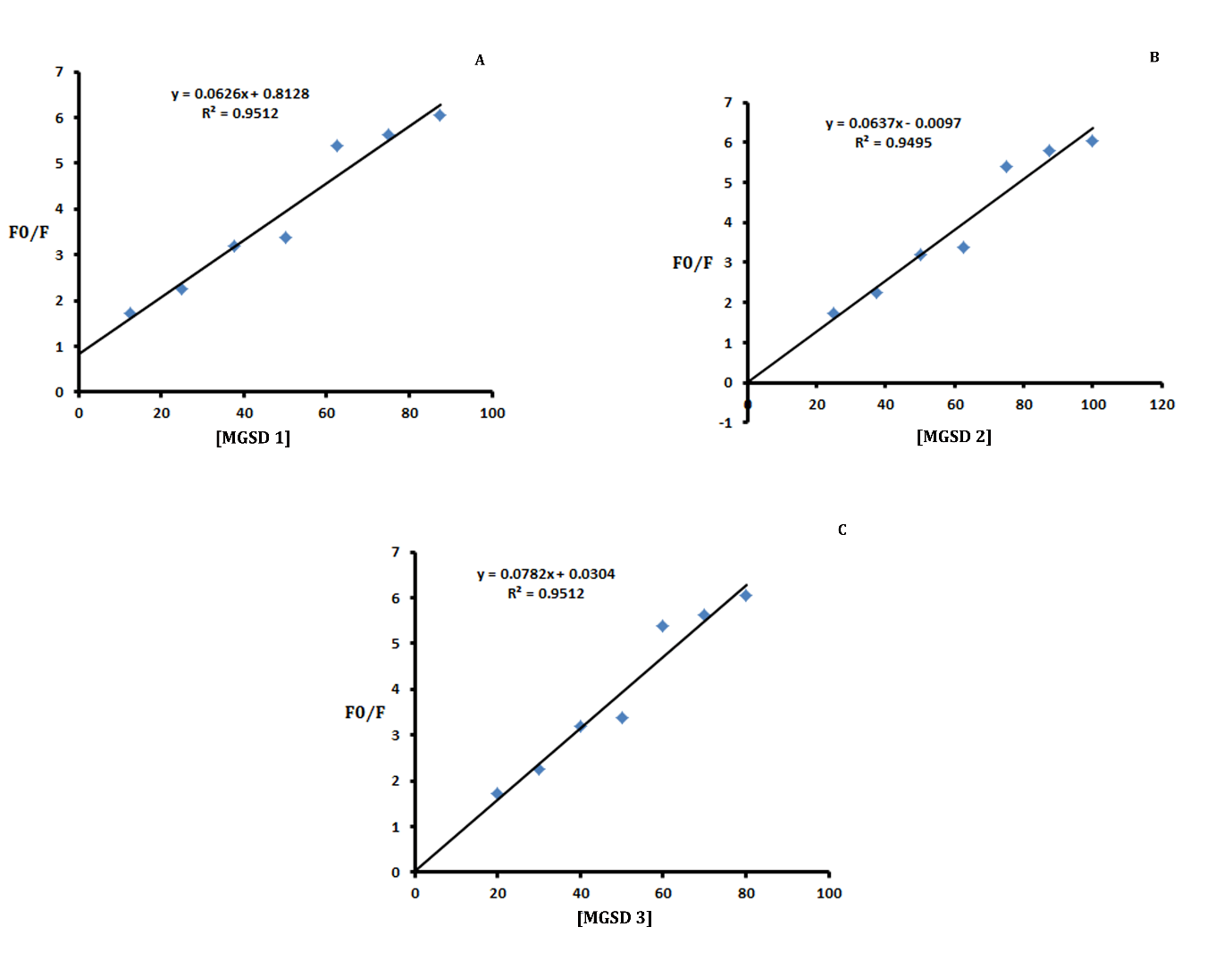


Supplementary Figure 15: Stern–Volmer plot between F_0_/F and [Q] [MGSD 1 (A), MGSD 2 (B) and MGSD 3 (C)]


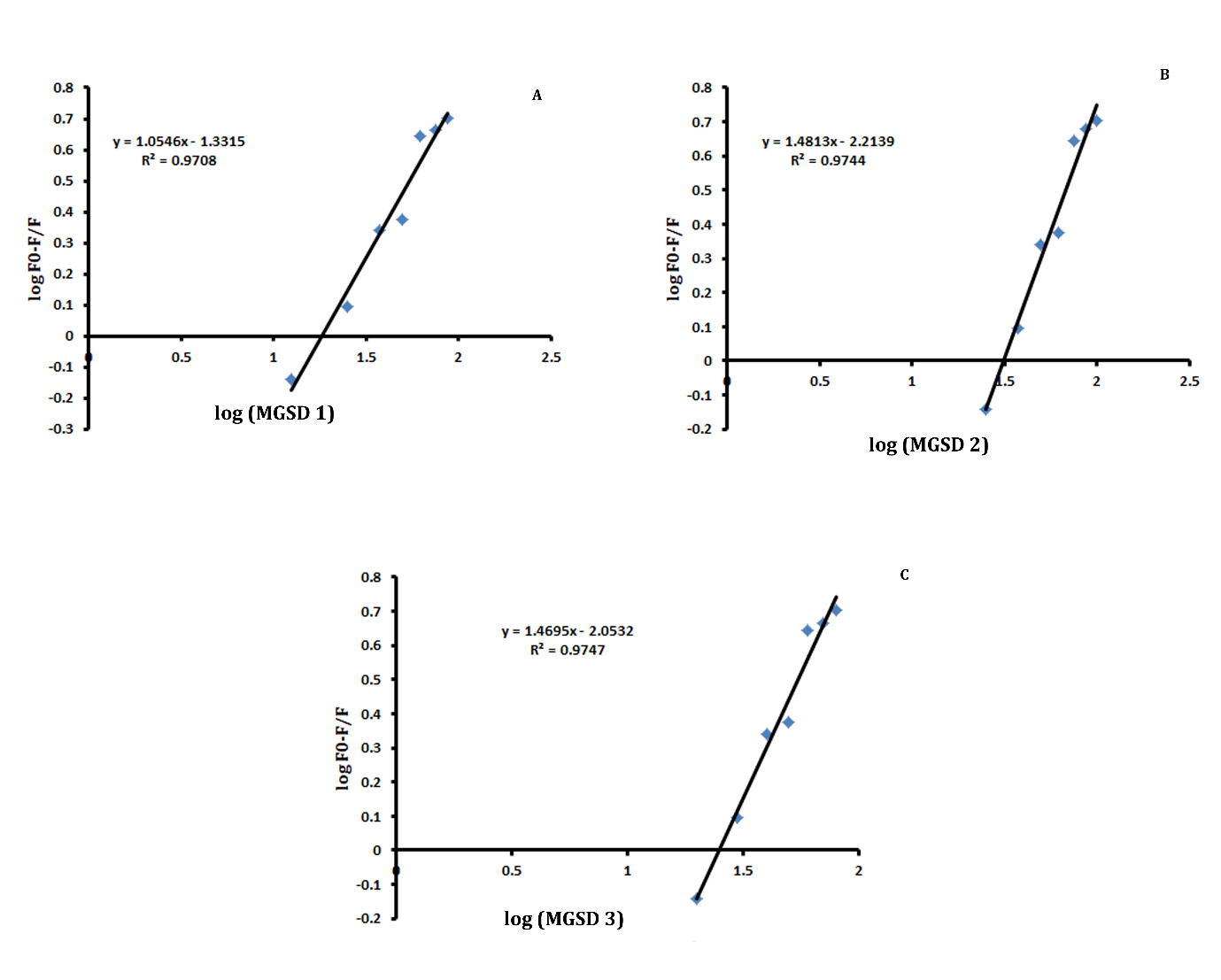


Supplementary Figure 16: The plot of log F_0_/F_0_-F vs log [Q] [MGSD 1 (A), MGSD 2 (B) and MGSD 3 (C)]

**Appendix I**

**Scheme 1:** Synthetic scheme of MGSD 1

**Scheme 1:** Synthetic scheme of MGSD 2 and MGSD 3

**Triethyl 2,2',2''-((5-(methoxycarbonyl) benzene-1,2,3-triyl) tris(oxy)) triacetate (compound 2):** To the solution of compound 1 (2.0 g, 10.86 mmol, 1.0 equiv) in DMF (20 mL) was added NaH (0.86 g, 35.86 mmol, 3.3 equiv). The reaction mass was cooled to 0°C and was added bromo ethyl acetate (6.0 g, 32.58 mmol, 3.0 equiv) drop wise and stirred the reaction mass at room temperature for 2 h. To the reaction mass was cooled to 0°C and quenched with ice water (100 mL). The reaction mass was extracted with ethyl acetate (3 x 100 mL). The organic layers were combined, dried over sodium sulphate, filtered and concentrated under reduced pressure. The crude product was purified using column chromatography using 15% ethyl acetate in hexane to get compound 2 (1.1 g, 23% yield) as white solid.

**2,2',2''-((5-carboxybenzene-1,2,3-triyl)tris(oxy))triacetic acid (MGSD 1):** The compound **2** (1.0 g) was taken in conc. HCl (10 mL) and heated to 100 ^o^C for 24 h. The reaction mass was cooled to room temperature and white solid was precipitated. The solid was filtered and dried to get **MGSD 1** (0.3 g, 38 % yield) as white solid. **^1^H NMR** (400 MHz, DMSO-*d6*): δ 12.93 (bs, 3H), 7.14 (s, 2H), 4.78 (s, 4H), 4.66 (s, 2H); **LCMS:** *m/z* calculated for C_13_H_12_O_11_: 344.04; Observed mass: 344.90 (M+1); CHN analysis (C, 47.46; H, 3.76) (Supplementary Figure 7 & 11).

**Diethyl 2,2'-((3-hydroxy-5-(methoxycarbonyl)-1,2-phenylene)bis(oxy))diacetate (compound 3) and methyl 3-(2-ethoxy-2-oxoethoxy)-4,5-dihydroxybenzoate (compound 4):** To the solution of compound 1 (2.0 g, 10.86 mmol, 1.0 equiv) in DMF (20 mL) was added Cesium carbonate (10.58 g, 32.58 mmol, 3.0 equiv). The reaction mass was cooled to 0°C and was added bromo ethyl acetate (4.0 g, 21.72 mmol, 2.0 equiv) drop wise and stirred the reaction mass at room temperature for 2h. To the reaction mass was cooled to 0°C and quenched with water (100 mL). The reaction mass was extracted with ethyl acetate (3 x 100 mL). The organic layers were combined, dried over sodium sulphate, filtered and concentrated under reduced pressure. The crude product was purified using column chromatography using 50% ethyl acetate in hexane to get compound **3** (0.6 g, 16 % yield) as white solid and using 100% ethyl acetate to get compound **4** (0.6 g, 20 % yield).

**2,2'-((5-carboxy-3-hydroxy-1,2-phenylene)bis(oxy))diacetic acid (MGSD 2):**. The compound **3** (0.6 g) was taken in conc. HCl (5 mL) and heated to 100 oC for 24 h. The reaction mass was cooled to room temperature and white solid was precipitated. The solid was filtered and dried to get compound **MGSD 2** (0.2 g, 40 % yield) as white solid. **^1^H NMR** (400 MHz, DMSO-*d6*): δ 7.13 (s, 1H), 6.99 (s, 1H), 4.87 (s, 2H), 4.67 (s, 2H); **LCMS:** *m/z* calculated for C_13_H_12_O_11_: 286.03; Observed mass: 287.2 (M+1); CHN analysis (C, 46.28; H, 3.63) (Supplementary Figure 8, 9 &12).

**3-(carboxymethoxy)-4,5-dihydroxybenzoic acid (MGSD 3):** The compound **4** (0.6 g) was taken in conc. HCl (5 mL) and heated to 100 ^o^C for 24 h. The reaction mass was cooled to room temperature and white solid was precipitated. The solid was filtered and dried to get **MGSD 3** (0.2 g, 40 % yield) as white solid. **^1^H NMR** (400 MHz, DMSO-*d6*): δ 7.11 (s, 1H), 6.93 (s, 1H), 5.82 (bs, 1H), 5.59 (s, 1H), 4.73 (s, 2H); **LCMS:** *m/z* calculated for C_9_H_8_O_7_: 228.03; Observed mass: 227.2 (M-1); CHN analysis (C, 45.39; H, 3.45) (Supplementary Figure 10 &13).
